# A stacking ensemble with Pareto optimization for scalable electricity theft detection via hybrid data repair and lightweight deployment

**DOI:** 10.1038/s41598-026-39693-z

**Published:** 2026-03-23

**Authors:** Mohammed Ateequr Rahaman, Rasyidah Mohamad Idris

**Affiliations:** Faculty of Electrical Engineering, Universiti Teknologi Malaysia, Johor Bahru, Johor Malaysia

**Keywords:** Electricity theft detection, Smart meter analytics, Stacking ensemble learning, Multi-objective optimization, Model interpretability, Lightweight deployment, Energy science and technology, Engineering, Mathematics and computing

## Abstract

Electricity theft poses a significant challenge to grid reliability and utility revenues, while its detection using smart meter data is constrained by data quality issues, severe class imbalance, and practical deployment limitations. This study presents a stacking ensemble framework, termed Scalable Trustworthy Lightweight Network (STL-Net), for electricity theft detection (ETD) using smart meter data. The proposed framework integrates hybrid data repair, class imbalance handling, and temporal dimensionality reduction with a heterogeneous stacking ensemble composed of NGBoost, CatBoost, LightGBM, and XGBoost. Hyperparameters of the base learners are optimized using the Non-dominated Sorting Genetic Algorithm II (NSGA-II) to identify Pareto-optimal configurations that jointly consider predictive performance and model complexity before stacking. Model interpretability is supported through SHapley Additive exPlanations (SHAP), which provide transparent analysis of detection outcomes. Experiments conducted on real-world smart meter data demonstrate that STL-Net achieves an ROC-AUC of 0.9869 and an F1-score of 94.47%, outperforming a wide range of machine learning, ensemble, and deep learning baselines across multiple evaluation metrics. A lightweight variant, STL-Lite, preserves comparable detection performance (ROC-AUC: 0.9858) while reducing inference latency by approximately 40%, making it suitable for resource-constrained environments. These results indicate that the proposed framework effectively integrates accuracy, computational efficiency, and interpretability for ETD in smart grid applications.

**Supplementary Information:**

The online version contains supplementary material available at 10.1038/s41598-026-39693-z.

## Introduction and literature review

### Problem significance and scope

 Electricity theft remains a major source of non-technical losses (NTLs) worldwide, arising from unauthorized consumption, meter bypassing, or deliberate manipulation of metering records. It imposes severe financial pressure on utilities, with global losses estimated at approximately USD 101.2 billion annually, and developing economies such as India accounting for USD 17 billion in NTLs^1^. Beyond revenue erosion, theft distorts demand profiles and compromises operational planning by biasing load forecasting and asset scheduling, thereby increasing technical stress across distribution networks^2^. These distortions increase the risk of voltage instability, feeder congestion, and transformer overloading, which can propagate local faults into wider service disruptions and reduce overall grid reliability^3^. In regions where infrastructure is already constrained, such disruptions further intensify energy access challenges by increasing outage frequency and prolonging restoration times, with downstream implications for economic activity and sustainable development^4^.

The widespread deployment of advanced metering infrastructure (AMI) has reshaped this problem space while also intensifying these challenges. While AMI provides high-resolution consumption measurements and enables scalable analytics, it also expands the attack surface for both cyber and physical interference, including tampering, communication disruption, and degradation of meter data integrity^5–9^. At the same time, operational deployments must contend with imperfect field data, where missing readings, corrupted records, and inconsistent sampling can weaken model reliability and inflate misclassification costs. These realities limit the effectiveness of inspection-driven and rule-based strategies in large-scale, data-rich settings^10^.

Consequently, electricity theft detection (ETD) systems must operate reliably in the presence of data imperfections, sustain detection performance under severe class imbalance, and meet the low-latency requirements imposed by real-world deployment constraints. The following section reviews existing ETD paradigms and outlines the corresponding design requirements for scalable detection frameworks.

### Existing detection paradigms

Electricity theft detection (ETD) strategies are broadly categorized into hardware-based and data-driven (non-hardware) approaches, each offering distinct advantages and limitations in mitigating non-technical losses across modern power systems. Hardware-based methods rely on physical interventions, including tamper-resistant meters equipped with magnetic or optical sensors, real-time line monitoring using current transformers, and sealed enclosures incorporating radio-frequency identification mechanisms^11,12^. These solutions provide localized protection in high-risk areas but are often costly to deploy at scale, with per-unit installation costs reported in the range of USD 120–200^13^. In addition to economic constraints, hardware-centric approaches face scalability challenges in geographically dispersed or resource-limited regions and are vulnerable to communication failures in harsh or disaster-prone environments^14,15^. As a result, their applicability is typically restricted to targeted deployments rather than system-wide monitoring.

In contrast, data-driven ETD approaches leverage the growing availability of smart meter data and advanced analytics to identify anomalous consumption behavior. Early non-hardware methods include rule-based systems, clustering, and statistical thresholding, which are computationally efficient but rely on static behavioral assumptions that fail to capture evolving theft strategies such as partial bypassing or time-dependent manipulation^16–18^. More recent work has adopted machine learning (ML) techniques, including supervised classifiers, ensemble methods, and deep learning architectures, to model complex consumption patterns and improve detection accuracy^19–22^. However, complex architectures such as convolutional neural network–long short-term memory (CNN–LSTM) models often incur substantial computational and memory overheads, with reported inference latencies and resource requirements that limit their suitability for real-time, edge-level deployment^23–26^.

This contrast highlights a persistent gap in ETD research: hardware-based solutions lack cost-effective scalability, while purely data-driven approaches often trade deployment feasibility and transparency for predictive performance. These limitations motivate the development of hybrid, lightweight, and interpretable frameworks that can operate reliably on imperfect data while meeting the latency and resource constraints of real-world smart grid deployments.

### Persistent challenges in machine learning for electricity theft detection

Despite advances in machine learning (ML), electricity theft detection (ETD) systems continue to face deployment barriers that limit scalability, robustness, and operational trust. These challenges are tightly coupled and often compound one another, particularly under real-world conditions characterized by noisy data, severe class imbalance, and resource-constrained deployment environments.

Recent studies have explored unsupervised clustering, robust principal component analysis (PCA), semi-supervised learning, and supervised ensemble classifiers to improve detection performance^27–29^. While such approaches yield promising results under controlled conditions, they often address individual challenges in isolation, reducing their effectiveness when confronted with concurrent data imperfections and deployment constraints.

#### Missing data and adversarial missingness

Smart meter datasets commonly contain 12–40% missing values due to sensor faults, communication failures, or deliberate tampering^30,31^. In ETD contexts, missingness often follows a missing-not-at-random (MNAR) mechanism, where data gaps correlate with fraudulent activity, leading to distorted consumption profiles and reduced detection sensitivity^32,33^.

Conventional imputation methods, including deletion, mean substitution, and linear interpolation, introduce bias and fail to preserve temporal structure^34,35^. Time-series techniques such as autoregressive integrated moving average (ARIMA) improve continuity but cannot capture multivariate dependencies^36–38^. Probabilistic approaches, including Gaussian mixture models and matrix factorization, are constrained by distributional assumptions and limited adaptability^39,40^.

Recent advances in machine learning (ML) have enhanced the effectiveness of imputation methods in smart grid contexts. Ensemble regressors, such as XGBoost and MissForest, reconstruct theft-related anomalies by learning multivariate relationships. However, they often lack robustness against adversarial missingness, where data gaps are deliberately introduced to evade detection^41,42^. Hybrid strategies that integrate multiple imputation by chained equations (MICE) with neighborhood-based or boosting models have been explored to enhance robustness; however, they are typically applied as standalone preprocessing stages, which increases pipeline complexity and inference latency^43,44^. Deep learning–based approaches, such as generative adversarial networks (GANs) and transformer models, enable high-fidelity reconstruction of temporal consumption profiles. However, their use is associated with increased computational requirements and additional data handling considerations in large-scale smart metering environments^45,46^. These limitations motivate the consideration of imputation strategies that are robust, efficient, and tightly integrated with downstream detection objectives^47–50^.

#### Class imbalance: the Long-Tail barrier in ETD

ETD datasets are inherently imbalanced, with theft cases often representing less than 10% of observations^51^. This imbalance biases classifiers toward majority-class predictions, inflating accuracy while masking undetected theft incidents^52^. As a result, metrics such as recall, F1-score, ROC-AUC, and Matthews correlation coefficient (MCC) are more appropriate indicators of detection reliability.

Basic resampling techniques suffer from well-known drawbacks, including overfitting in oversampling and information loss in undersampling^53,54^. Synthetic methods such as the synthetic minority oversampling technique (SMOTE) and adaptive synthetic sampling (ADASYN) generate minority instances but may introduce unrealistic temporal artifacts^55^. Cost-sensitive learning requires accurate cost matrices, which are rarely available in dynamic grid environments^56^, while ensemble-based balancing methods introduce additional computational overhead^57^.

Deep generative approaches and one-class classifiers have been explored for handling highly imbalanced ETD scenarios; however, their applicability is influenced by training data requirements, computational overhead, and sensitivity to subtle variations in consumption behaviour^58,59^. Moreover, concept drift—arising from evolving fraud strategies—can rapidly degrade model performance without adaptive mechanisms^60–63^. Recent ETD studies have investigated advanced learning strategies to mitigate imbalance-induced bias, including cost-sensitive ensemble learning and representation-aware classification frameworks that improve minority-class separability under scarce theft samples^64,65^. These challenges underscore the need for imbalance-aware strategies that balance detection sensitivity with computational feasibility.

#### High dimensionality and feature redundancy

High-frequency smart meter measurements generate thousands of features per consumer, which increases computational burden and weakens model generalization when irrelevant or noisy variables are present^66–70^. Simple statistical summaries are computationally efficient but fail to represent nonlinear and temporal consumption dynamics^71^. Dimensionality reduction methods, including principal component analysis (PCA), compress feature space but often sacrifice interpretability^72,73^. Deep representation learning introduces substantial latency and resource demands that limit feasibility for edge-level deployment^74–76^.

Recent work advocates hybrid feature engineering strategies that combine temporal compression with information-theoretic selection to retain discriminative features while controlling model complexity^77^. Such approaches offer a practical balance between efficiency, interpretability, and detection accuracy.

#### Classifier Selection, Ensembles, and interpretability

Classifier selection plays a critical role in ETD, as it determines the balance between discrimination capability, robustness, and computational feasibility. Commonly used classifiers in ETD, including decision tree–based methods, support vector machines (SVM), and Random Forests (RF), differ in their transparency, scalability, and robustness to class imbalance^78–84^. To improve generalization, ensemble learning strategies—including bagging, boosting, and stacking—have been widely adopted to aggregate complementary decision patterns across multiple learners^85–93^. Probabilistic ensemble variants, such as NGBoost, further extend this capability by providing uncertainty-aware predictions that are valuable in ambiguous or borderline theft cases^94^.

However, increased model complexity often comes at the expense of transparency, limiting trust and acceptance in operational and regulatory settings. Explainable artificial intelligence techniques, particularly SHapley Additive exPlanations (SHAP), provide feature-level attribution and support accountability in ETD systems^92,94^. Despite these advances, most existing approaches treat interpretability as a post hoc analysis rather than integrating it within an end-to-end detection framework.

### Research gaps

Despite notable advancements in machine learning (ML)-based electricity theft detection (ETD), several persistent challenges continue to impede real-world deployment in smart grid environments. A primary gap arises from the fragmented design of existing ETD pipelines. Current studies typically address co-occurring data challenges—such as missingness through imputation (e.g., MICE, KNN, XGBoost)^31–33^, class imbalance via SMOTE-Tomek or related variants^54,62^, and high dimensionality using PCA or kernel PCA^73^—in isolation. Likewise, explainability techniques such as SHapley Additive exPlanations (SHAP) are most often applied post hoc^23^, rather than being integrated into the learning pipeline. However, these stages interact in complex, nonlinear ways: suboptimal imputation can distort minority-class boundaries, which may propagate through resampling and classifier training, ultimately degrading detection accuracy. The literature still lacks cohesive, end-to-end frameworks that jointly address hybrid data repair, multi-objective optimization, and regulatory-grade interpretability^23,31,54,73^.

A second limitation concerns computational feasibility and scalability. Many advanced ETD models, including deep learning–based architectures and large ensemble classifiers such as CNN–LSTM hybrids and gradient-boosted trees, achieve strong predictive performance but incur high inference latency, commonly ranging from 300 to 500 ms per prediction^22,23,25^. This latency substantially exceeds the practical constraints reported for real-time or near-real-time deployment on resource-constrained edge devices embedded within smart meters^24,25^. As a result, deployment feasibility remains limited, especially in resource-constrained or distributed grid environments.

Third, although stacking ensembles are increasingly adopted in ETD research, they are often evaluated only against individual base learners or generic classifiers. Comparative benchmarking against alternative state-of-the-art stacking configurations is rarely performed, and the trade-offs between marginal accuracy gains, inference latency, and deployment cost are seldom examined. This limits clarity regarding when stacking architectures provide meaningful practical advantages beyond incremental performance improvements.

Further challenges relate to generalizability and robustness. Most ETD models are trained on region-specific datasets with high AMI penetration, which restricts their applicability in low-resource settings^5,13^. In parallel, evolving theft strategies—such as remote tampering and stealthy consumption masking—introduce risks of model degradation when encountering previously unseen behaviours^56,72^. Emerging cyber-physical threats, including data poisoning and evasion attacks, further complicate ETD pipelines, yet remain largely unaddressed in conventional workflows^6,13,16^.

Finally, many existing optimization strategies in ETD prioritize predictive accuracy as a primary objective, while comparatively less attention is given to computational efficiency and architectural simplicity^23,49^. This emphasis can lead to overprovisioned hardware requirements and inefficient resource utilization, limiting feasibility at scale.

Collectively, these gaps highlight the need for an integrated ETD framework that can simultaneously:

(i) address hybrid data imperfections involving missingness and imbalance,
(ii) manage high-dimensional feature spaces without compromising interpretability,
(iii) achieve Pareto-efficient trade-offs between accuracy, latency, and model complexity, and.
(iv) embed transparency as a core design requirement to support regulatory compliance and operational trust.

### Proposed framework (STL-Net)

To address the persistent challenges in electricity theft detection (ETD), including missing data, class imbalance, high-dimensional feature spaces, limited interpretability, and deployment constraints, this study proposes STL-Net, a Scalable, Trustworthy, and Lightweight Network. STL-Net is formulated as an end-to-end pipeline that integrates data repair, class balancing, feature engineering, ensemble learning, and explainability within a unified and deployment-aware architecture.

Specifically, the framework incorporates multiple imputation by chained equations (MICE), *k*-nearest neighbours (KNN), and gradient boosting–based regression for hybrid data repair; SMOTE-Tomek for class rebalancing; piecewise aggregate approximation (PAA) for temporal compression; and mutual information (MI) for feature relevance assessment, with full methodological details provided in Sect. “Feature selection using mutual information”.

To manage the high dimensionality of high-frequency consumption data, STL-Net performs temporal compression followed by relevance-based feature selection, resulting in a reduced feature space while retaining interpretability and predictive capability. This processing allows downstream models to operate on compact and informative representations that are compatible with large-scale deployment.

The classification core of STL-Net is a heterogeneous stacking ensemble comprising NGBoost, CatBoost, LightGBM, and XGBoost as base learners, with an XGBoost meta-learner. Base learner hyperparameters are optimized using the Non-dominated Sorting Genetic Algorithm II (NSGA-II) to identify Pareto-efficient configurations that balance detection performance with computational complexity before stacking.

STL-Net integrates SHAP to provide global and instance-level explanations of model decisions without introducing inference overhead. In addition, a lightweight deployment variant, STL-Lite, is introduced to support resource-constrained environments by reducing model size and inference latency while maintaining comparable detection performance. The components of STL-Net and their correspondence to key ETD challenges are summarized in Supplementary Table (ST1).

### Contributions

This study makes the following contributions to electricity theft detection (ETD):

1. **Unified End-to-End Framework**: A scalable and deployment-aware ETD framework, termed STL-Net, is proposed. Unlike existing pipelines that address data repair, imbalance handling, optimization, and interpretability in isolation, STL-Net integrates these components within a single end-to-end architecture.
2. **Pareto-Optimized Stacking Ensemble for ETD**: A heterogeneous stacking ensemble is developed using NGBoost, CatBoost, LightGBM, and XGBoost as base learners, optimized via NSGA-II. The optimization explicitly balances detection performance against computational complexity and inference latency, addressing practical deployment constraints often overlooked in prior ETD studies.
3. **Robust Handling of Data Imperfections**: The framework incorporates hybrid data imputation, adaptive class rebalancing, temporal compression, and relevance-based feature selection to maintain reliable performance in the presence of missing data, severe class imbalance, and high-dimensional consumption profiles.
4. **Deployment-Oriented Lightweight Variant**: A compact variant, STL-Lite, is introduced to support resource-constrained environments. By simplifying the ensemble architecture and applying model compression, STL-Lite achieves reduced inference latency and memory footprint while maintaining comparable detection performance.
5. **Integrated Model Interpretability**: Feature-level interpretability is embedded using SHAP to provide global and instance-level explanations, supporting transparent decision-making and regulatory accountability in real-world utility operations.
6. **Comprehensive Evaluation and Benchmarking**: The proposed framework is evaluated using extensive benchmarking against individual classifiers, deep learning models, and alternative stacking configurations, employing multiple performance and efficiency metrics to assess both detection accuracy and deployment feasibility.

## Proposed methodology

### Overview of the STL-Net framework

**Fig. 1:**
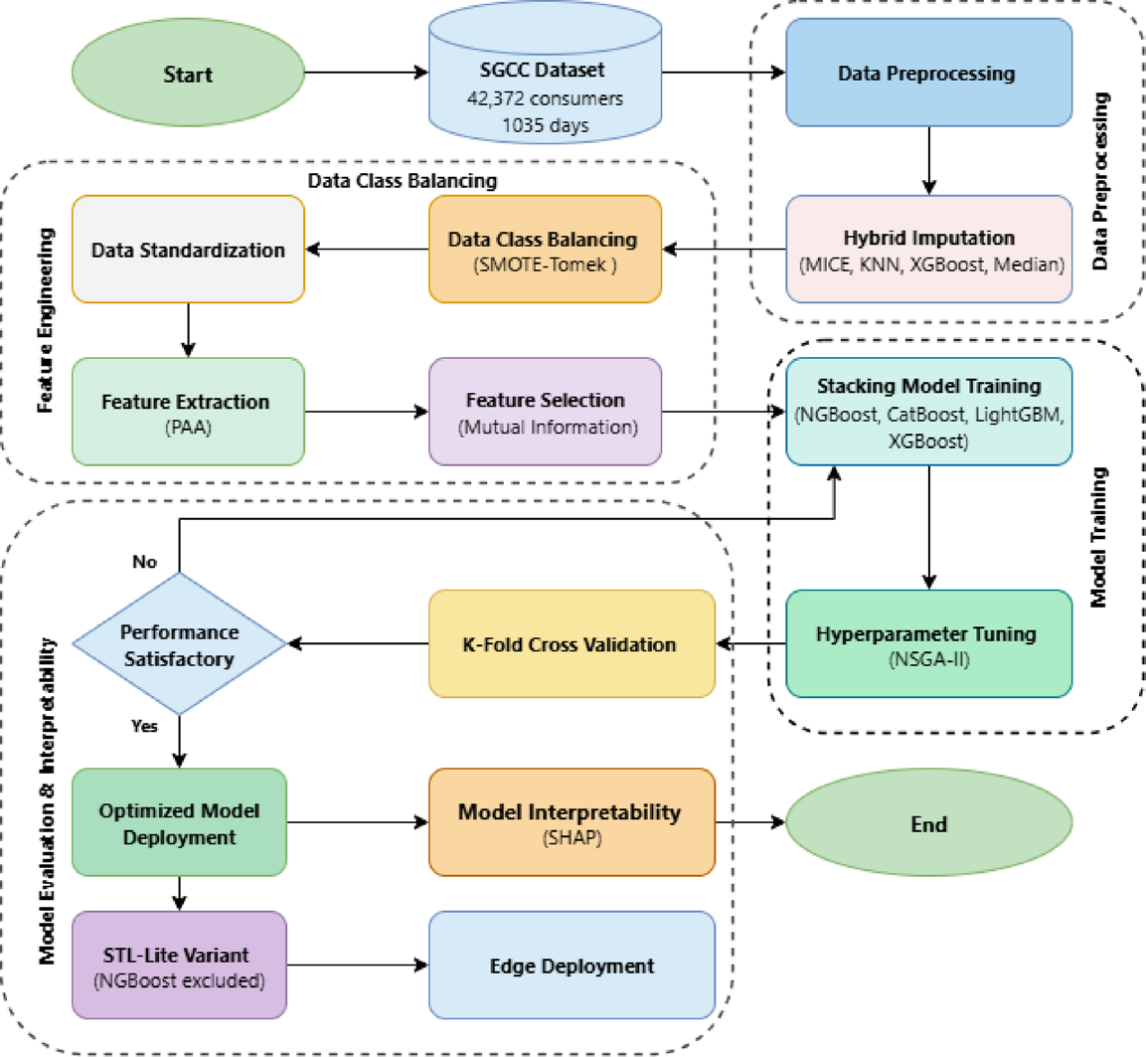
Conceptual STL-Net workflow showing leakage-safe hybrid imputation, data class balancing, feature engineering, Pareto-optimized stacking, and the STL-Lite latency-oriented variant.

This section presents the proposed Scalable Trustworthy Lightweight Network (STL-Net) framework for electricity theft detection (ETD). STL-Net is designed as an end-to-end, deployment-aware pipeline that jointly addresses data imperfections, class imbalance, high dimensionality, model optimization, and interpretability within a unified architecture. In contrast to conventional ETD pipelines that treat preprocessing, classification, and explanation as independent stages, STL-Net accounts for the dependencies between these components by implementing them as a leakage-safe, sequential pipeline. Figure 1 illustrates the overall workflow of STL-Net, highlighting the sequential integration of data preparation, feature engineering, ensemble optimization, and deployment-oriented inference.

The framework integrates hybrid data repair, imbalance-aware learning, compact feature representation, Pareto-optimized stacking, and explainable decision analysis to support reliable detection under real-world operational constraints. Conceptually, the framework comprises data preprocessing, feature engineering and class balancing, ensemble training and optimization, and interpretability-aware deployment, while retaining flexibility in implementation.

### Dataset description and preprocessing

This study utilizes a publicly available dataset provided by the State Grid Corporation of China (SGCC), which contains daily electricity consumption records over 1,035 consecutive days (January 2014 to October 2016)^74^. The dataset includes 42,372 labeled consumers, with a class distribution of 91.46% genuine and 8.54% fraudulent. Table 1 summarizes the dataset’s key metadata, including sampling period and class distribution.

**Table 1:** Metadata description of the SGCC dataset.

Description	Value
Consumption Period	January 2014 – October 2016 (1035 days)
Total consumers	42,372
Genuine consumers	38,757 (91.46%)
Fraudulent consumers	3,615 (8.54%)
Total records	43,855,020

To prevent information leakage, the dataset was first split into training and test sets using stratified sampling. All preprocessing steps—including hybrid imputation, SMOTE-Tomek resampling, PAA transformation, mutual-information feature selection, and model training—were fitted exclusively on the training data (or within training folds during cross-validation). The held-out test partition was not used during preprocessing or model selection and was retained for final evaluation. Within each training fold, imputation and scaling were fitted first, followed by SMOTE–Tomek resampling, then PAA transformation, MI-based feature selection, and model fitting.

To qualitatively examine consumption behaviour, representative daily load profiles of fraudulent and genuine consumers were analyzed. Fraudulent-labelled consumers often display low or irregular usage episodes and intermittent surges, whereas genuine-labelled consumers exhibit comparatively smoother periodic patterns; representative examples are shown in Supplementary Figures (SF1–SF2).

### Missing data analysis and hybrid imputation strategy

Smart meter datasets often contain missing readings due to sensor faults, communication failures, and field-level disruptions. In ETD contexts, missingness may additionally coincide with suspicious or adversarial behaviour, increasing the risk of distorted consumption profiles and reduced detection sensitivity if not handled appropriately. An exploratory analysis of missing values revealed heterogeneous sparsity patterns across consumers, with most records exhibiting low to moderate missingness and a smaller subset showing pronounced gaps indicative of localized data corruption. The distribution of missing values across consumer records is illustrated in Supplementary Figure (SF3).

To address this challenge, STL-Net employs a sequential hybrid imputation pipeline designed to preserve temporal structure while accommodating varying levels of sparsity. The pipeline consists of four stages:

i. Multiple Imputation by Chained Equations (MICE) for highly sparse features.
ii. *k*-Nearest Neighbours (KNN) imputation for moderately sparse features.
iii. XGBoost-based regression refinement to model nonlinear dependencies.
iv. Final median adjustment to ensure numerical stability.

A 30% missingness threshold was used to distinguish highly sparse from moderately sparse features. This value was selected based on an empirical coverage analysis across all consumers, which indicated a transition in data availability around this range. Sensitivity analysis conducted over thresholds from 10% to 50% missingness demonstrated that the 30% cutoff provides a favorable trade-off between retained consumers and downstream detection performance, particularly in terms of ROC-AUC and F1-score. The robustness of this threshold choice is supported by comparative results reported in Supplementary Table (ST2) and Supplementary Figure (SF4).

The hybrid imputation design ensures that each component operates under conditions best suited to its underlying assumptions: MICE captures multivariate dependencies in highly incomplete features, KNN preserves local similarity structures for moderately sparse attributes, and XGBoost refines residual inconsistencies by modeling nonlinear temporal consumption patterns. The final median-based adjustment stabilizes feature distributions before downstream learning.

A summary of the progressive refinement of missing, zero, and invalid values across imputation stages is reported in Supplementary Table (ST3), while the impact of logarithmic transformation on reducing distributional skewness is quantified in Supplementary Table (ST4). Detailed mathematical formulations, parameter settings, and additional sensitivity analyses are provided in Supplementary Note (SN1) to support methodological transparency and reproducibility.

### Class imbalance handling and feature engineering

Electricity theft detection (ETD) datasets are inherently imbalanced, with fraudulent consumers representing a small minority of the overall population. In the SGCC dataset, only 8.54% of consumers are labeled as fraudulent, creating a strong bias toward majority-class predictions if not explicitly addressed. Such an imbalance can inflate accuracy while masking undetected theft cases, making imbalance-aware learning essential for reliable ETD.

#### Adaptive class rebalancing using SMOTE–Tomek

To mitigate class imbalance while preserving decision boundary integrity, STL-Net adopts the SMOTE–Tomek strategy. This hybrid approach combines the Synthetic Minority Oversampling Technique (SMOTE) with Tomek Links to simultaneously enhance minority-class representation and remove ambiguous majority-class samples near class boundaries. SMOTE generates synthetic minority instances by interpolating between each minority sample and its *k* nearest minority neighbours, improving recall without duplicating observations. Tomek Links then removes the majority of samples involved in nearest-neighbour cross-class pairs, reducing overlap and cleaning borderline regions.

The neighbour parameter *k* controls the locality of synthetic sample generation and is dataset-dependent; therefore, STL-Net uses *k* = 5 and verifies robustness through a sensitivity check over *k* ∈ {3, 5, 7, 9} within the training folds. All resampling operations were applied exclusively within training folds to prevent information leakage, and the original class distribution of the test set was preserved for unbiased evaluation. The effect of class rebalancing on class distribution and feature-space separability is illustrated in Supplementary Figures (SF6–SF7)**.**

#### Temporal compression via piecewise aggregate approximation

High-frequency smart meter data give rise to high-dimensional feature spaces, which substantially increase computational demands and can adversely affect model generalization. To address this, STL-Net applies Piecewise Aggregate Approximation (PAA) to compress daily consumption profiles into lower-dimensional representations. PAA partitions each time series into fixed-length segments and replaces each segment with its mean value, retaining dominant consumption trends while suppressing short-term fluctuations.

The number of PAA segments was selected based on prior smart-meter analytics literature and practical deployment considerations, to retain meaningful temporal structure while controlling feature dimensionality and computational overhead. In this study, each 1,035-day consumption profile is compressed into 50 PAA features, corresponding to an average window length of approximately 20–21 days per segment. This configuration represents a pragmatic trade-off between temporal resolution and representation compactness for downstream electricity theft detection tasks. An illustrative comparison between raw standardized consumption profiles and their PAA representations is provided in Supplementary Figure (SF8). A brief sensitivity checks on the number of PAA segments (30, 50, and 70) is reported in Supplementary Note (SN2), indicating that 50 segments provide stable ROC-AUC while reducing runtime.

#### Feature selection using mutual information

Following temporal compression, Mutual Information (MI) is employed to quantify nonlinear dependencies between each feature and the target label. Features contributing limited discriminatory information are removed, yielding a compact and informative feature set. This two-stage feature engineering process—temporal compression followed by relevance-based selection—ensures that downstream classifiers operate on compact and interpretable representations with reduced computational overhead. The mutual information ranking of PAA-derived features and the selected subset is shown in Supplementary Figure (SF9). Detailed mathematical formulations, parameter settings, and diagnostic analyses for class balancing and feature engineering are provided in Supplementary Note (SN2).

### Stacking model training and optimization

To overcome the limitations of individual classifiers and enhance robustness in electricity theft detection (ETD), this study adopts a stacking ensemble learning strategy tailored to large-scale, imbalanced smart meter data. The stacking framework integrates multiple heterogeneous base learners with a meta-learner, facilitating the aggregation of complementary decision behaviors and improving generalization relative to single-model classifiers. Within the proposed STL-Net framework, stacking is employed not merely to improve predictive performance but as a structured mechanism to balance detection accuracy, computational efficiency, and deployment feasibility. All model training and hyperparameter optimization procedures were conducted exclusively on the training data following the leakage-safe protocol described in Sect. “Dataset description and preprocessing”. Stratified cross-validation was employed within the training set to preserve class proportions and mitigate overfitting during optimization and ensemble construction.

#### Stacking model architecture

Stacking is an ensemble learning strategy that combines the outputs of multiple heterogeneous base learners through a higher-level meta-learner to improve predictive robustness and generalization. By aggregating complementary decision patterns learned by diverse model architectures, stacking is particularly effective for complex and imbalanced classification problems such as electricity theft detection (ETD).

**Fig. 2:**
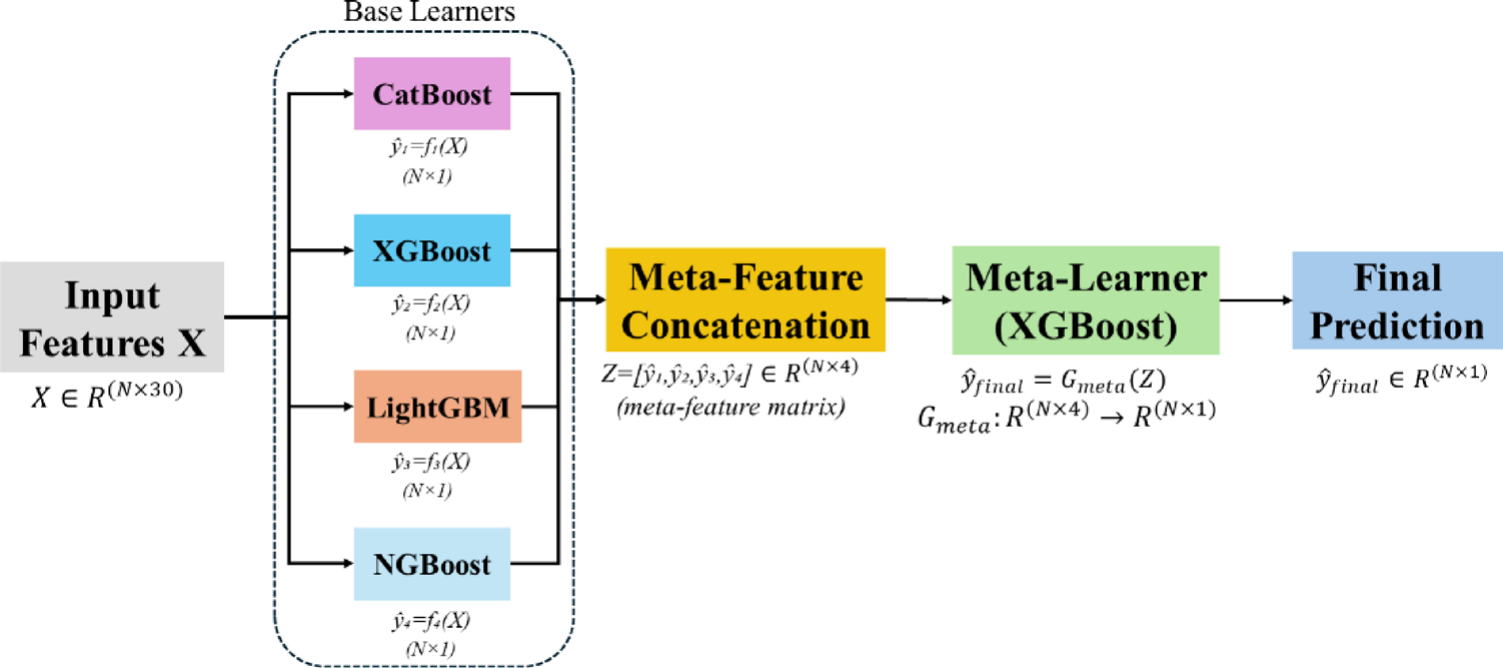
STL-Net stacking architecture illustrating MI-selected inputs, heterogeneous base learners, and meta-learner fusion for theft probability prediction.

Figure 2 presents the STL-Net stacking architecture. Following preprocessing, temporal compression, and mutual information (MI)–based feature selection, the model receives an input feature matrix $\:X\in\:{\mathrm{R}}^{N\times\:30}$, where *N* denotes the number of consumers, and 30 represents the selected features. Four heterogeneous base learners—CatBoost, XGBoost, LightGBM, and NGBoost—are trained independently on *X* to estimate the probability of electricity theft for each sample. Each base learner produces a scalar probability score for the theft class, and these outputs are concatenated to form a meta-feature matrix $\:Z\in\:{\mathrm{R}}^{N\times\:4}$. An XGBoost meta-learner subsequently maps *Z* to the final theft probability.

The selection of heterogeneous base learners is motivated by their complementary strengths. CatBoost effectively captures complex feature interactions, XGBoost provides strong performance on structured tabular data, LightGBM offers computational efficiency for large-scale datasets, and NGBoost supplies probabilistic predictions that enhance uncertainty awareness. Their integration enables STL-Net to reduce model bias and improve robustness relative to any individual learner.

Formally, let $\:X=\{{x}_{1},{x}_{2},\dots\:,{x}_{N}\}$denote the input feature matrix with corresponding labels $\:y=\{{y}_{1},{y}_{2},\dots\:,{y}_{N}\}$. Each base learner $\:{f}_{k}$, $\:k\in\:\{\mathrm{1,2},\mathrm{3,4}\}$, produces a probability estimate for the theft class according to Eq. (1):

1 $$\:\widehat{{y}_{k}}\:=\:{f}_{k}\left(X\right),\:\:k\in\:\:\left\{1,\:2,\:3,\:4\right\}$$

where $\:\widehat{{y}_{k}}$ represents the fraud probability estimated by the *k*-th base model.

The outputs of the base learners are concatenated to form the meta-feature matrix *Z* shown in Eq. (2), which serves as input to the meta-learner.

2 $$\:Z=[{\widehat{y}}_{1},{\widehat{y}}_{2},{\widehat{y}}_{3},{\widehat{y}}_{4}]$$

The meta-learner *G* is then trained on *Z* with true labels *y*, producing the final fraud probability as shown in Eq. (3):

3 $$\:{\widehat{y}}_{final}=G\left(Z\right)=G({f}_{1}\left(X\right),\:{f}_{2}\left(X\right),\:{f}_{3}\left(X\right),\:{f}_{4}\left(X\right))$$

where $\:{\widehat{y}}_{final}$ represents the final fraud probability after model stacking.

This stacked formulation allows STL-Net to exploit complementary predictive signals across base learners while maintaining a compact and deployment-efficient inference pipeline.

#### Multi-Objective hyperparameter optimization using NSGA-II

Electricity theft detection (ETD) models exhibit a practical trade-off between discriminative performance and computational complexity. STL-Net characterizes this trade-off using Pareto-based multi-objective optimization (NSGA-II) to obtain base learners that balance ROC-AUC and complexity under a leakage-safe inner validation protocol. Rather than treating hyperparameter tuning as a purely performance-driven task, the proposed STL-Net framework positions the Non-Dominated Sorting Genetic Algorithm II (NSGA-II) as a scientific instrument for discovering, analyzing, and exploiting the fundamental accuracy–complexity frontier governing ETD models.

To test this hypothesis, NSGA-II was employed to optimize the hyperparameters of each base learner within the stacking ensemble under a bi-objective formulation. For a given hyperparameter configuration *H*, the optimization objectives were defined using Eqs. (4) and (5):

4 $$\:\mathrm{Maximize}:\:{f}_{1\:}\left(H\right)=\mathrm{A}\mathrm{U}\mathrm{C}\:\left(H\right)$$

5 $$\:\mathrm{Minimize}:\:{f}_{2}\:\left(H\right)=\:C\left(H\right)$$

where AUC(*H*) is the area under the ROC curve estimated on validation data, and using equations (6a) and (6b), where *C(H)* represents model complexity. For tree-based learners (CatBoost, XGBoost, and LightGBM), model complexity was defined as a weighted sum of tree depth and number of boosting estimators:

6a $$\:C\left(H\right)=\:\alpha\:\cdot\:depth+\:\beta\:\cdot\:estimators\:$$

whereas for NGBoost, which does not expose a tree-depth parameter, complexity was defined solely by the number of estimators:

6b $$\:C\left(H\right)=\beta\:\cdot\:estimators\:$$

To ensure numerical comparability between objectives, both $\:{f}_{1}$and $\:{f}_{2}$were normalized to the range [0,1] range using min–max scaling within each NSGA-II population. This normalization prevents dominance of either objective during Pareto sorting and enables stable convergence of the evolutionary search.

A sensitivity analysis was conducted on a stratified validation subset (15% of the training data) to assess the influence of the weighting coefficients *α* and *β* across the range [0.1,2.0]. Equal weighting (*α = β = 1)* consistently produced stable Pareto fronts and interpretable trade-off structures and was therefore adopted throughout the study.

To ensure reproducibility and prevent information leakage, NSGA-II was executed within an inner optimization loop using only the training data. All preprocessing operations—including hybrid imputation, feature scaling, SMOTE-Tomek class rebalancing, PAA-based temporal compression, and mutual-information-based feature selection—were fitted exclusively on the inner training folds before optimization. The held-out test set was not accessed during optimization and was reserved solely for final performance evaluation.

The hyperparameter search spaces were defined based on library documentation and prior empirical studies. NSGA-II was configured with a population size of 60 over 20 generations, using simulated binary crossover (_c_ = 0.9) and polynomial mutation (_m_ = 0.2). Early stopping was triggered when the hypervolume improvement remained below 1% for five consecutive generations. All optimization experiments were implemented using the *pymoo* framework^71–73^.

From the resulting Pareto-optimal solution sets, final hyperparameter configurations were selected using a knee-point criterion, defined as the region of the Pareto front where marginal gains in ROC-AUC diminish sharply relative to increases in model complexity. This selection strategy yields models that are neither accuracy-dominated nor computationally overprovisioned, aligning with real-world deployment constraints. The selected knee-point configurations for all base learners are summarized in Table 2 and were subsequently fixed for constructing and evaluating both the full STL-Net ensemble and its lightweight deployment variant.

**Table 2:** Optimized hyperparameters for base learners in the stacking ensemble.

Model	Learning Rate	Depth	Estimators
CatBoost	0.30	12	69
XGBoost	0.28	9	150
LightGBM	0.30	8	100
NGBoost	0.29	–	199

In this manner, NSGA-II functions not merely as a tuning mechanism but as an analytical tool that reveals the fundamental accuracy–efficiency frontier governing ETD models^95^.

#### STL-Lite variant for deployment efficiency

To enable practical deployment in latency-sensitive grid environments, a lightweight variant of the proposed STL-Net framework, referred to as STL-Lite, was developed. STL-Lite was obtained by excluding NGBoost, identified as the most computationally intensive base learner during profiling experiments. Measurements conducted in the Google Colab Pro environment revealed that NGBoost incurred an average inference latency of approximately 12.4 ms per record, compared to 3.1–4.6 ms for XGBoost, CatBoost, and LightGBM. This substantial latency made NGBoost unsuitable for real-time ETD scenarios, particularly in resource-constrained edge infrastructures where low-millisecond latency is preferred for near–real-time screening.

STL-Lite retained XGBoost, CatBoost, and LightGBM as base learners while preserving the same preprocessing pipeline, meta-feature generation strategy, and stacking structure as the full STL-Net model. Hyperparameters optimized via NSGA-II (Table 2) were reused without re-tuning to maintain methodological consistency and enable fair performance comparison.

Inference time was measured using Python’s ‘*timeit*’ module, averaged over 100 runs. Excluding NGBoost reduced the ensemble’s average inference latency by approximately 40%, without compromising classification performance. The XGBoost meta-learner was retained to maintain structural integrity, though its contribution to runtime is acknowledged. Future work may explore replacing the meta-learner with a simpler classifier, such as logistic regression, to reduce inference time. Overall, STL-Lite offers a robust and resource-efficient solution for real-time deployment of ETD systems in smart grids, addressing computational and latency constraints while maintaining the high accuracy of the full STL-Net architecture.

#### Baseline stacked ensemble models for comparative evaluation

To enable a fair and methodologically transparent comparison with existing ensemble-based electricity theft detection (ETD) approaches, three representative stacked ensemble baselines (denoted S1–S3) were implemented following configurations commonly reported in recent ETD literature. These baselines were designed to reflect established stacking strategies rather than to replicate the full optimization pipeline proposed in STL-Net.

Specifically, S1 represents a classical stacking framework composed of Random Forest, Extra Trees, and Gradient Boosting as base learners, with Logistic Regression as the meta-learner. This configuration reflects early and widely adopted stacking formulations in ETD studies, where linear meta-models are used to combine heterogeneous tree-based predictors. S2 corresponds to a non-linear stacking architecture employing heterogeneous tree-based base learners with XGBoost as the meta-learner, representing more recent ETD approaches that emphasize accuracy-oriented ensemble fusion. S3 reflects an alternative literature-inspired stacking design commonly used in ETD studies, in which ensemble composition and fusion rules are selected heuristically to maximize predictive accuracy under fixed computational budgets.

All stacked baselines were trained using the same preprocessing pipeline, feature set, data splits, and stratified 10-fold cross-validation protocol as the proposed STL-Net framework. Importantly, NSGA-II was not applied to S1–S3 by design. Hyperparameters for the baseline stacks were selected using standard validation-based tuning or commonly reported defaults, consistent with their treatment in prior ETD literature. Applying multi-objective Pareto optimization to the baselines would effectively transform them into variants of the proposed framework, thereby obscuring the methodological distinction this study seeks to evaluate.

This design choice intentionally preserves a clear methodological contrast: while S1–S3 represent conventional stacking practices optimized primarily for predictive accuracy under single-objective or heuristic tuning, STL-Net uniquely integrates Pareto-based multi-objective optimization to jointly balance detection performance, model complexity, and deployment feasibility. Consequently, performance differences observed in Sect. “Comparative evaluation of model performance ” can be attributed to the proposed optimization-aware stacking strategy rather than to variations in data handling, feature engineering, or evaluation protocol. Notably, even when strong accuracy-oriented stacking baselines (e.g., S2) achieve competitive performance, STL-Net demonstrates consistent gains in recall, agreement metrics (MCC and Cohen’s kappa), and robustness under deployment-relevant constraints, justifying the necessity of the proposed framework beyond accuracy-only stacking designs.

### Stacking model evaluation

The proposed STL-Net stacking ensemble was evaluated using a structured and leakage-safe methodology to assess its accuracy, generalization ability, interpretability, and deployment feasibility. Evaluation was conducted for both the full STL-Net architecture and the streamlined STL-Lite variant to enable deployment-oriented comparisons. Hyperparameter optimization via NSGA-II was completed prior to evaluation using only training data, and the held-out test set was not used during tuning or model selection. Five complementary evaluation components were employed: stratified cross-validation for stability analysis, confusion matrix–based metrics, AUC–ROC analysis, SHAP-based interpretability, and system-level runtime assessment.

#### K-Fold Cross-Validation

To assess performance stability and mitigate variance under class imbalance, STL-Net was evaluated using 10-fold stratified cross-validation. The dataset was partitioned into ten folds while preserving class proportions. In each iteration, one fold was used for validation, and the remaining nine folds for training. This process was repeated across all folds. Mean performance across folds was reported to provide a robust estimate of model consistency.

All evaluated models used hyperparameters fixed via NSGA-II optimization (Sect. “Multi-objective hyperparameter optimization using NSGA-II”). Cross-validation scores were computed using Eq. (7).

7 $$\:{CV}_{score}=\:\frac{1}{k}\:\sum\:_{i\:=1}^{k}{Score}_{i}$$

where *k* is the number of folds and *Score*_*i*_ denotes the performance metric for the *i*^*th*^ fold.

#### Confusion matrix evaluation

The confusion matrix provides a detailed breakdown of classification outcomes, including true positives (TP), false positives (FP), true negatives (TN), and false negatives (FN). These quantities were used to compute multiple evaluation metrics, defined in Eqs. (8)- (13):

8 $$\:Accuracy=\:\frac{TP+TN}{TP+TN+FP+FN}$$

9 $$\:Precision=\:\frac{TP}{TP+FP}$$

10 $$\:Recall\:=\:\frac{TP}{TP+FN}$$

11 $$\:F1-score=2\:\times\:\:\frac{Precision\:\times\:Recall}{Precision+Recall}$$

12 $$\:MCC=\:\frac{\left(TP\:\times\:TN\right)-(FP\:\times\:FN)}{\sqrt{\left(TP+FP\right)\left(TP+FN\right)\left(TN+FP\right)(TN+FN)}}$$

13 $$\:\kappa\:=\:\frac{{P}_{0}-{P}_{e}}{1-\:{P}_{e}}$$

Here, *P*_*0*_ is the observed agreement, and *P*_*e*_ is the expected agreement by chance. While accuracy is reported for completeness, imbalance-aware metrics such as F1-score, MCC, and Cohen’s kappa are emphasized due to the skewed class distribution inherent in ETD datasets. These metrics were computed for both STL-Net and STL-Lite, supporting a direct trade-off assessment between accuracy and inference speed.

#### AUC-ROC curve

Receiver Operating Characteristic (ROC) curves were used to evaluate the discrimination capability of the models across decision thresholds by plotting the true positive rate (TPR) against the false positive rate (FPR). The area under the ROC curve (AUC) provides a threshold-independent measure of separability, which is particularly suitable for imbalanced classification problems such as ETD. These metrics are defined in Eqs. (14)–(15).

14 $$\:TPR=\:\frac{TP}{TP+FN}$$

15 $$\:FPR=\:\frac{FP}{FP+TN}$$

#### Model interpretability via SHAP

Given the black-box nature of ensemble models, SHapley Additive exPlanations (SHAP) were employed to enhance interpretability. SHAP values quantify the contribution of each feature to individual predictions, enabling both global importance ranking and local decision analysis. Summary plots identify dominant drivers of theft detection, while dependence plots reveal nonlinear feature interactions. SHAP analysis was conducted for both STL-Net and STL-Lite to verify that the lightweight variant preserves interpretability and supports transparent, audit-ready decision-making in operational utility contexts.

#### Computational environment

All experiments were conducted on Google Colab Pro, utilizing an NVIDIA Tesla T4 GPU, a 2-core Intel Xeon CPU, and 25 GB of RAM. The complete STL-Net pipeline, including preprocessing, model training, and NSGA-II optimization, was executed in approximately 4.2 h. STL-Lite, which excludes NGBoost, achieved a 40% reduction in inference latency while maintaining accuracy within a negligible margin of the full model. These results indicate that both variants are computationally feasible under the reported CPU/GPU environment, with STL-Lite providing reduced inference latency under resource-constrained settings.

## Results and discussion

This section presents a comprehensive evaluation of STL-Net for electricity theft detection (ETD). Performance is first assessed using leakage-safe stratified cross-validation, followed by benchmarking against baseline machine learning models, deep learning architectures, and alternative stacking ensembles. Statistical significance testing is conducted to examine the consistency of observed improvements across folds. Additional analyses investigate convergence behaviour, inference latency, and model interpretability using SHAP. Deployment-oriented performance under the original class prevalence is evaluated separately using precision–recall–based metrics.

### K-Fold Cross-Validation analysis of the proposed model

The proposed stacking ensemble (STL-Net) was evaluated using 10-fold stratified cross-validation following the preprocessing and training protocol described in Sect. “Dataset description and preprocessing”. Each fold preserved the original class proportions, and model hyperparameters were fixed to the NSGA-II–optimized values reported in Sect. “Multi-objective hyperparameter optimization using NSGA-II”. Performance metrics were computed independently for each fold and subsequently averaged to obtain stable estimates of generalization performance.

Across the ten folds, STL-Net achieved a mean accuracy of 94.38%, precision of 92.85%, recall of 96.16%, F1-score of 94.47%, and ROC-AUC of 98.69%. Agreement-based measures further confirm robust classification behavior, with a Cohen’s kappa of 88.75% and an MCC of 88.81%. The standard deviation across folds remained below 0.4% for all metrics, indicating consistent performance under repeated resampling. These results demonstrate that the proposed stacking strategy yields stable and reliable predictive performance across multiple train–validation partitions. Detailed fold-wise results are provided in the Supplementary Table (ST5).

### Comparative evaluation of model performance

This subsection benchmarks STL-Net against its constituent base learners (before and after NSGA-II optimization), conventional machine learning models, deep learning architectures, and representative stacking ensemble baselines. Performance is first examined by comparing base learners before and after NSGA-II hyperparameter optimization, with performance differences assessed using paired t-tests and Wilcoxon signed-rank tests. STL-Net is then evaluated against conventional machine learning and deep learning models, followed by a comparison with alternative stacked ensemble configurations. Finally, deployment feasibility is examined using the STL-Lite variant, which prioritizes inference efficiency under latency-sensitive grid environments.

#### Performance benchmarking and impact of NSGA-II tuning

The performance of the proposed stacking ensemble (STL-Net) was benchmarked against its constituent base learners—XGBoost, LightGBM, CatBoost, and NGBoost—both before and after hyperparameter optimization via NSGA-II. The optimization strategy, objective formulation, and parameter search spaces are detailed in Sect. “Multi-objective hyperparameter optimization using NSGA-II”. All results reported in this subsection are averaged over a 10-fold stratified cross-validation to ensure robustness and fair comparison.

Table 3 summarizes the performance of the individual base learners and the default (untuned) stacking ensemble. Even without optimization, STL-Net outperformed all individual base learners, achieving 85.97% accuracy, 86.18% F1-score, and 93.24% ROC-AUC. Relative to the strongest untuned base learner (XGBoost), this corresponds to improvements of 1.11% points (pp) in accuracy and 0.94 pp in ROC-AUC, indicating that aggregating heterogeneous learners already provides measurable gains over standalone models.

**Table 3:** Performance of base classifiers and default ensemble before NSGA-II tuning.

Classifier	Accuracy (%)	Precision (%)	Recall (%)	F1-Score (%)	ROC-AUC (%)	Kappa (%)	MCC (%)
XGBoost	84.86	83.39	87.06	85.18	92.30	69.71	69.78
LightGBM	78.72	78.81	78.56	78.68	86.89	57.44	57.44
CatBoost	83.52	82.74	84.70	83.71	91.23	67.04	67.06
NGBoost	69.18	72.55	61.71	66.69	76.80	38.36	38.80
**STL-Net**	**85.97**	**84.91**	**87.49**	**86.18**	**93.24**	**71.94**	**71.97**

Table 4 reports performance after NSGA-II optimization. Consistent improvements are observed across all classifiers, confirming the effectiveness of Pareto-based tuning in navigating the trade-off between discrimination and model complexity. Among individual learners, optimized XGBoost achieves the highest ROC-AUC (98.21%), while CatBoost exhibits balanced precision–recall performance.

**Table 4:** Performance of base classifiers and the stacking ensemble after NSGA-II tuning.

Classifier	Accuracy (%)	Precision (%)	Recall (%)	F1-Score (%)	ROC-AUC (%)	Kappa (%)	MCC (%)
XGBoost	93.54	92.02	95.35	93.65	98.21	87.07	87.13
LightGBM	89.52	87.85	91.73	89.74	95.78	79.04	79.11
CatBoost	90.89	89.16	93.09	91.08	96.66	81.78	81.86
NGBoost	89.94	87.58	91.61	89.55	95.62	79.01	79.12
**STL-Net**	**94.38**	**92.85**	**96.16**	**94.47**	**98.69**	**88.75**	**88.81**

The optimized STL-Net, constructed by integrating all NSGA-II-tuned base learners with XGBoost as the meta-learner, achieved the strongest overall performance: 94.38% accuracy, 94.47% F1-score, and 98.69% ROC-AUC, alongside a Cohen’s kappa of 88.75% and MCC of 88.81%. Relative to the best untuned base learner, these results correspond to a 9.52 pp increase in accuracy, an 8.29 pp improvement in F1-score, and a 16.81 pp gain in Cohen’s kappa, reflecting improved inter-class agreement and reduced bias.

**Fig. 3:**
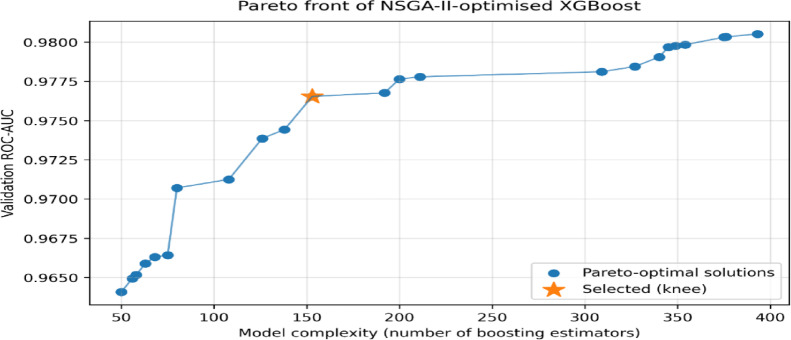
Pareto front obtained using NSGA-II for XGBoost, illustrating the trade-off between validation ROC-AUC and model complexity.

To further examine the behavior of the multi-objective optimization process underlying these improvements, Fig. 3 presents a representative Pareto front obtained using NSGA-II for XGBoost. Each point corresponds to a non-dominated hyperparameter configuration for which no alternative solution simultaneously achieves higher ROC-AUC and lower model complexity. The observed frontier reveals a clear accuracy–efficiency trade-off, where marginal gains in discrimination require increasingly higher computational cost. The final operating point was selected from the knee region of the Pareto front, providing a balanced compromise between predictive performance and complexity. Pareto fronts for the remaining base learners are provided in the Supplementary Figures (SF10 – SF11).

**Fig. 4:**
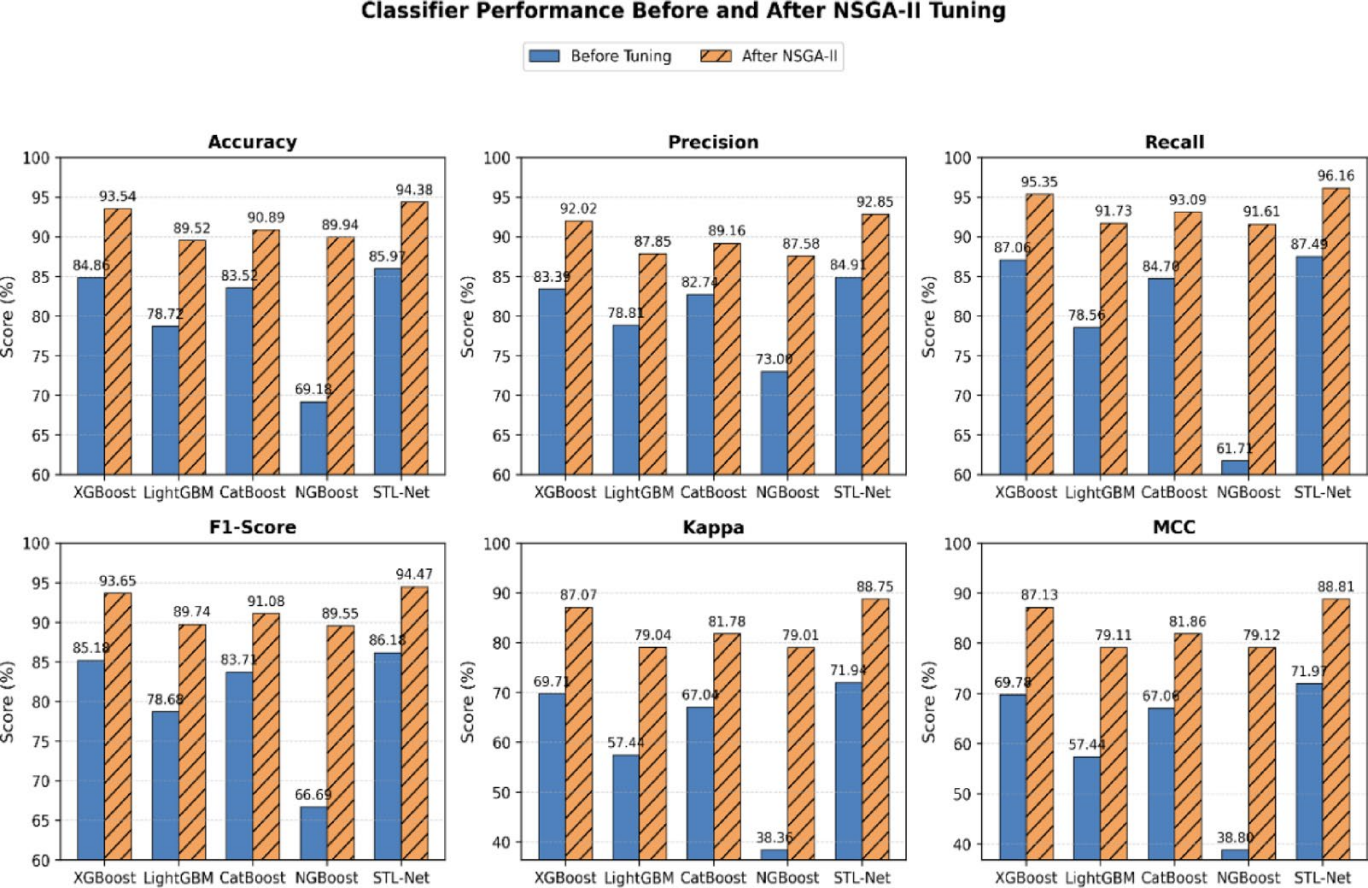
Comparative performance of base classifiers before and after NSGA-II tuning.

Using the selected Pareto-optimal configurations (Table 2), STL-Net was retrained and evaluated under the same cross-validation protocol. Figure 4 visualizes the comparative performance of classifiers before and after tuning, showing consistent improvements of approximately 9–10 pp in accuracy, recall, and Cohen’s kappa, with precision and F1-score exhibiting similarly notable gains. These results demonstrate that the observed improvements arise from both multi-objective tuning and ensemble integration.

To isolate the contribution of the meta-learning stage, STL-Net was further compared against an unweighted average of the optimized base learners (Table 5). While simple averaging achieves an F1-score of 91.01% and ROC-AUC of 96.57%, STL-Net improves these to 94.47% and 98.69%, respectively. The corresponding gains of 3.46 pp in F1-score and 2.12 pp in ROC-AUC indicate that the meta-learner resolves residual errors and inter-model conflicts beyond what is achievable through aggregation alone. Overall, these results confirm that NSGA-II serves not merely as a tuning heuristic but as a mechanism for identifying balanced operating points on the accuracy–complexity frontier, and that stacking further exploits complementary decision patterns to enhance robustness in electricity theft detection.

**Table 5:** Comparative performance of the meta‑learner (STL‑Net) versus the average of base learners, with performance gains shown in percentage points.

Model	Accuracy (%)	Precision (%)	Recall (%)	F1-Score (%)	ROC-AUC (%)	Kappa (%)	MCC (%)
Avg. of Base Learners	90.97	89.65	92.95	91.01	96.57	81.98	82.06
STL-Net	94.38	92.85	96.16	94.47	98.69	88.75	88.81
**Performance Gain**	**+ 3.41**	**+ 3.20**	**+ 3.21**	**+ 3.46**	**+ 2.12**	**+ 6.77**	**+ 6.75**

#### Comparative evaluation with Baseline, deep Learning, and stacked ensemble models

To assess the effectiveness and practical relevance of the proposed STL-Net framework, its performance was compared against three categories of competing approaches: (i) conventional machine learning classifiers, (ii) deep learning architectures, and (iii) representative stacked ensemble baselines. All models were trained and evaluated under identical experimental conditions using stratified 10-fold cross-validation.

A complete numerical comparison across all baselines—including accuracy, precision, recall, F1-score, ROC-AUC, Matthews correlation coefficient (MCC), Cohen’s kappa, and average inference latency—is reported in the Supplementary Table (ST6). The discussion below focuses on the principal findings relevant to predictive performance and deployment feasibility.

Among conventional machine learning baselines, tree-based ensembles such as Extra Trees and Random Forest demonstrate relatively strong discrimination, achieving F1-scores above 91% and ROC-AUC values exceeding 97%. However, their performance remains consistently below that of STL-Net, particularly in recall, MCC, and Cohen’s kappa, indicating reduced robustness in capturing minority theft patterns and lower inter-class agreement.

Deep learning models, including CNN, LSTM, and CNN–LSTM architectures, exhibit lower predictive performance on the SGCC dataset, with F1-scores below 82% and substantially higher inference latency (≥ 12 ms per record). This behavior is consistent with prior observations that deep architectures offer limited advantage on structured, feature-engineered tabular data when temporal information has already been encoded through explicit feature extraction.

The strongest stacked ensemble baseline (S2) achieves competitive performance (ROC-AUC ≈ 0.984, F1-score ≈ 94.0%), confirming the effectiveness of ensemble integration. Nevertheless, STL-Net consistently surpasses all stacked baselines across accuracy, recall, F1-score, ROC-AUC, MCC, and Cohen’s kappa. Relative to the average performance of stacked baselines, STL-Net improves F1-score by more than 3% points and Cohen’s kappa by over 6% points, reflecting stronger discrimination and reduced classification bias.

In comparison, the stacked baselines rely on single-objective or heuristic tuning, whereas STL-Net uses Pareto-optimal hyperparameter selection via NSGA-II to model the performance–complexity trade-off. This design yields an ensemble configuration that achieves the strongest overall predictive performance while maintaining moderate CPU-only inference latency (approximately 6 ms per record).

**Fig. 5:**
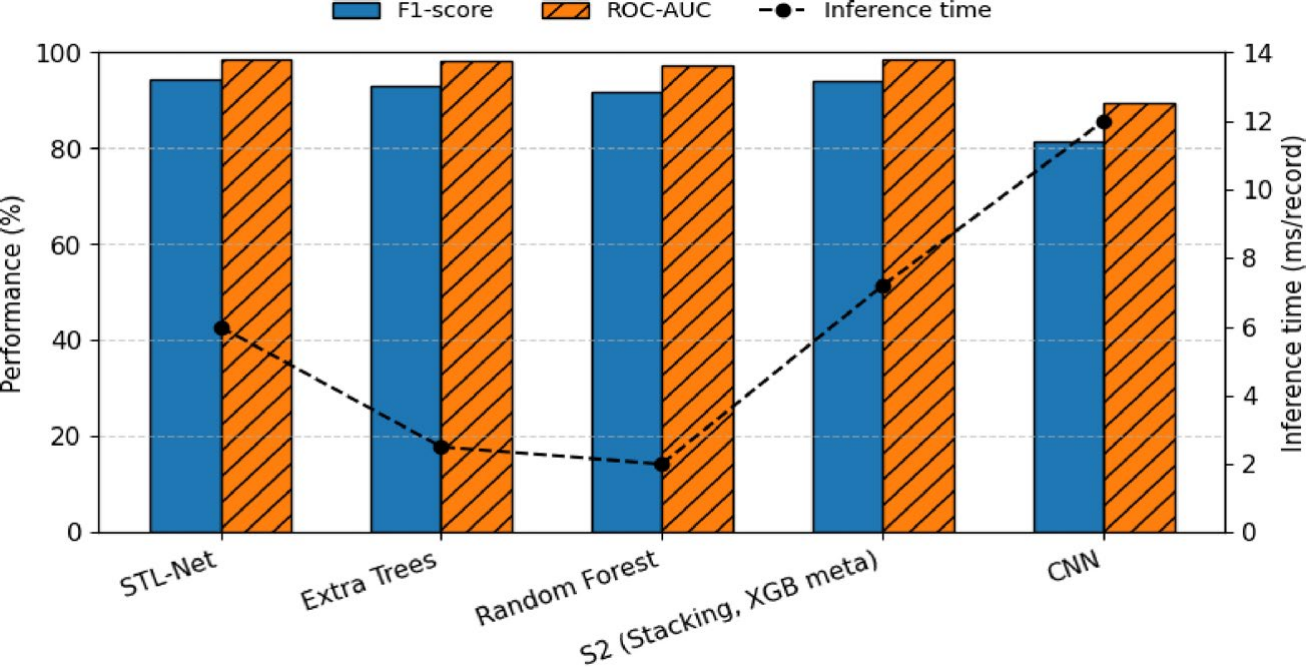
Comparative performance of the proposed STL-Net and representative baseline models on the SGCC dataset (10-fold stratified cross-validation). Bars show F1-score and ROC-AUC (left Y-axis), while the dashed line indicates average CPU inference latency per record (right Y-axis).

Figure 5 visualizes the comparative performance of the strongest baseline models and the proposed STL-Net, jointly illustrating predictive performance and inference efficiency. STL-Net attains the highest F1-score (94.5%) and ROC-AUC (98.7%) among all evaluated approaches while remaining suitable for real-time deployment. Complete numerical results for all evaluated models are provided in Supplementary Table.

#### Statistical significance testing and performance gains

To determine whether the observed performance gains of the proposed stacking ensemble (STL-Net) over competing models are statistically reliable, formal hypothesis testing was conducted using fold-wise results from stratified 10-fold cross-validation (*n* = 10). Pairwise comparisons were performed between STL-Net and each baseline classifier for three primary performance metrics: accuracy, F1-score, and ROC–AUC.

For each comparison, two-sided paired *t*-tests were applied to fold-wise metric differences, treating cross-validation folds as paired observations. As cross-validation sample sizes are limited, the robustness of the parametric results was further assessed using the Wilcoxon signed-rank test, which evaluates paired differences without relying on distributional assumptions.

Because multiple pairwise tests were conducted (14 baselines × 3 metrics = 42 tests), family-wise error was controlled using Bonferroni correction, yielding an adjusted significance threshold of α* ≈ 0.00119. Under this correction, all paired *t*-tests produced *p*-values < 1 × 10⁻⁵. Consistently, the Wilcoxon signed-rank test returned *p* = 0.00195 in cases where fold-wise differences uniformly favored STL-Net, supporting the same directional conclusion as the paired *t*-tests.

To quantify the magnitude of improvements, absolute performance gains Δ = (STL-Net − baseline) were computed in percentage points (pp), together with 95% confidence intervals for accuracy differences derived from fold-wise paired estimates. The smallest performance gaps were observed against strong tree-based ensembles. For example, when compared with Extra Trees, STL-Net improves accuracy by 0.38 pp, F1-score by 1.50 pp, and ROC–AUC by 0.39 pp. Against Random Forest, gains increase to 2.68 pp in accuracy and 2.70 pp in F1-score. Substantially larger margins are observed for linear and deep learning baselines, with accuracy gains exceeding 21 pp against CNN–LSTM and more than 36 pp against Naïve Bayes.

Complete numerical results—including fold-wise means, confidence intervals, Bonferroni-adjusted significance levels, and Wilcoxon *p*-values for all baselines, including stacked ensemble variants (S1–S3)—are reported in Supplementary Table (ST7). Overall, these findings demonstrate that the performance improvements achieved by STL-Net are consistent across folds, statistically robust under strict family-wise error control, and unlikely to be attributable to random variation.

#### Comparative analysis of Convergence, implementation Complexity, and error budget

To complement aggregate performance metrics, this subsection examines the proposed STL-Net framework and its base learners along three dimensions: convergence behavior, implementation complexity, and error budget characteristics. These analyses provide insight into the optimization dynamics and robustness underlying the performance gains reported in Sect. “Performance benchmarking and impact of NSGA-II Tuning”–“Statistical significance testing and performance gains”.

**Fig. 6:**
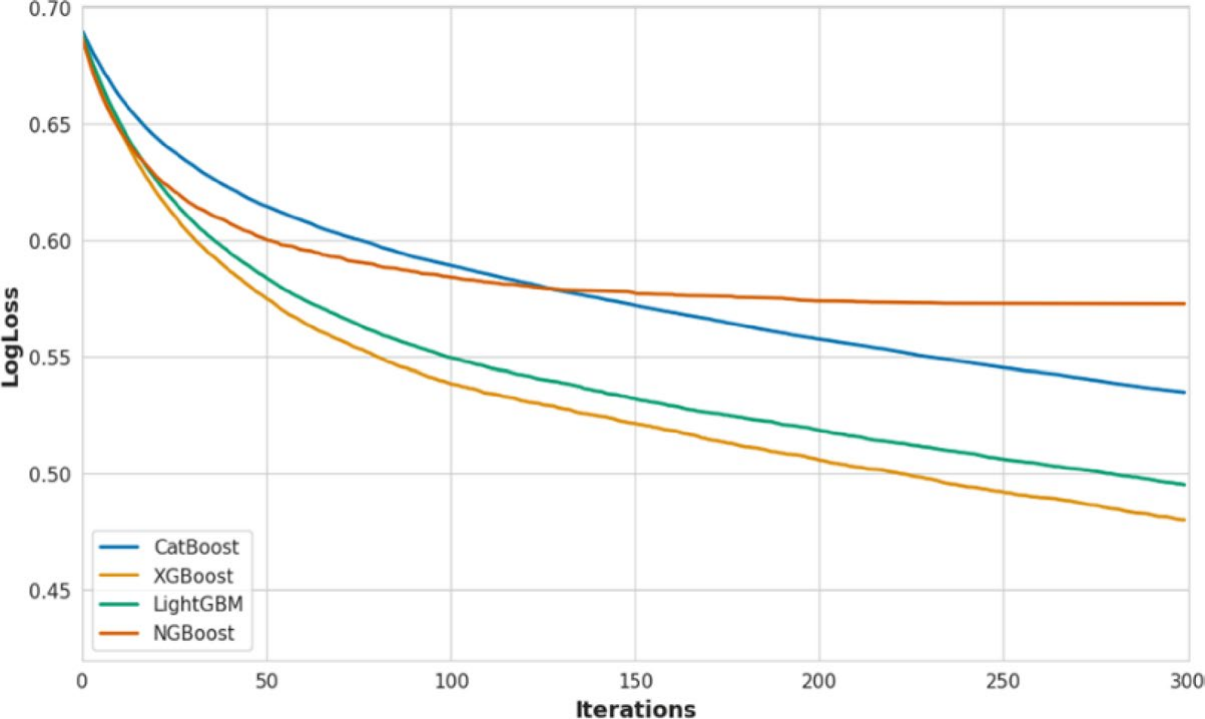
Log-loss convergence curves of optimized base classifiers over 300 boosting iterations.

Figure 6 presents the log-loss convergence profiles of the optimized base learners over 300 boosting iterations. XGBoost exhibits the fastest loss decay and achieves the lowest final log-loss, indicating efficient optimization. LightGBM follows closely, with comparable convergence behavior. NGBoost converges rapidly during early iterations but plateaus after approximately 150–200 rounds at a higher loss level. While CatBoost shows a slower but steady reduction in loss and remains in a descending regime at 300 iterations. Although CatBoost continues to improve beyond this range, NSGA-II consistently selected earlier knee-point configurations (Table 2), as additional iterations yielded diminishing performance gains relative to increased model complexity. STL-Net is not shown in Fig. 6 because its training is governed by meta-level learning under stratified cross-validation rather than a single iterative loss trajectory.

Implementation complexity and runtime characteristics, including convergence time, iterations to plateau, and tuning effort, are summarized in Supplementary Table (ST8). STL-Net incurs higher training cost due to sequential optimization of multiple base learners and meta-learning; however, this overhead is offset by the substantial improvements in generalization and stability demonstrated in Sect. “Comparative evaluation with baseline, deep learning, and stacked ensemble models” and “Statistical significance testing and performance gains”.

To further characterize predictive robustness, the total prediction error was decomposed into bias- and variance-related components using cross-validated estimates (Supplementary Table (ST9). Among individual learners, XGBoost and LightGBM exhibit balanced bias–variance trade-offs, while NGBoost shows higher total error. STL-Net achieves the lowest total error, reflecting an improved bias–variance balance enabled by heterogeneous model aggregation and meta-level learning.

#### Evaluation of STL-Lite (lightweight variant of STL-Net)

To enhance operational feasibility in real-time or resource-constrained environments, a lightweight variant of the STL-Net (denoted STL-Lite) was developed by excluding NGBoost, identified as the most computationally intensive base learner. The streamlined architecture retains XGBoost, CatBoost, and LightGBM as base models while preserving the original ensemble training pipeline, including meta-feature generation via stratified 10-fold cross-validation and XGBoost as the meta-learner.

Under identical preprocessing, training, and evaluation protocols, STL-Lite achieved 94.7% accuracy, class-wise F1-scores of 94.5% (non-theft) and 95.0% (theft), and 98.6% ROC-AUC (Table 6). These results closely match the full STL-Net (94.4% accuracy; 98.7% ROC-AUC), indicating that discriminative performance is largely preserved despite removing NGBoost.

**Table 6:** Comparative performance of STL-Net and STL-Lite (10-fold stratified CV). Class “0” = non-theft; class “1” = theft.

Model	Accuracy (%)	Precision (0/1) (%)	Recall (0/1) (%)	F1-Score (0/1) (%)	ROC-AUC (%)	Inference Time (ms/record)
STL-Net	94.4	93.0/93.0	96.0/96.0	94.0/94.0	98.7	6.0
STL-Lite	94.7	95.0/94.0	94.0/96.0	94.5/95.0	98.6	3.9

Efficiency improved materially: total training time decreased by 24%, and average CPU inference latency improved to 3.9 ms/record (from 6.0 ms/record for STL-Net). STL-Lite is therefore faster than all evaluated deep learning baselines (CNN: 12.0 ms; LSTM: 18.0 ms; CNN-LSTM: 25.0 ms; see Table (ST6), while maintaining competitive accuracy and AUC. STL-Lite is designed to reduce inference latency and model complexity, making it suitable for latency-sensitive deployment scenarios under the evaluated CPU-based setup.

#### Robustness and stability analysis

The proposed framework was evaluated under multiple stress conditions to assess reliability beyond standard train–test performance. The evaluation considered temporal drift, severe class imbalance, and feature perturbations, reflecting common sources of performance degradation in real-world electricity theft detection (ETD) deployments. As summarized in Table 7, STL-Net maintained stable ranking performance across all evaluated scenarios, with ROC–AUC remaining above 0.99 under temporal drift and exhibiting only limited variation under noise and imbalance stress. In contrast, threshold-dependent metrics, including accuracy and F1-score, were more sensitive to changes in operating conditions, indicating that observed performance variation is primarily associated with threshold sensitivity rather than degradation of ranking capability. Under the original, highly imbalanced class distribution, precision–recall analysis provided a more informative characterization of deployment behavior by reflecting the trade-off between inspection coverage and yield.

**Table 7:** Consolidated summary of robustness evaluations under adversarial test conditions.

Condition	Key Impact on Fraud Detection	ROC-AUC Stability	Overall Verdict
Class Imbalance	Large recall/F1 degradation without rebalancing; CatBoost showed the smallest degradation; NGBoost showed the largest.	Moderate (~ 0.77)	Requires Rebalancing
Temporal Distribution Shift	46 pp recall drop; macro F1 − 0.10; indicates threshold misalignment	High (> 0.99; slight + Δ)	Strong (with recalibration)
Feature Noise (σ = 0.05)	Similar recall/F1 drop; minimal extra penalty beyond temporal effects	Very high (> 0.99; +Δ)	Very Strong (with recalibration)

A consolidated comparison of robustness outcomes for STL-Net and the deployment-oriented STL-Lite variant is reported in Table 7, illustrating the expected performance–latency continuum between the full and lightweight ensembles. The feature noise robustness exhibited behavior consistent with temporal drift. Ranking performance remained stable across perturbation levels, whereas threshold-dependent metrics were more sensitive to changes in operating conditions. Detailed results for individual robustness experiments, including noise perturbation levels and fold-wise breakdowns, are provided in Supplementary Tables (ST10–ST12) and Supplementary Note (SN3).

STL-Net maintains stable ranking performance under temporal drift and feature noise (ROC–AUC > 0.99) while remaining threshold-sensitive in these settings, warranting periodic retraining and calibration. Under extreme class imbalance, fraud detection degrades materially without rebalancing, reinforcing the need for class-aware training in deployment.

#### Cross-domain robustness evaluation

To assess robustness beyond the ETD domain, an additional evaluation was conducted on the Pima Indians Diabetes dataset^96^. This experiment does not claim domain transferability; rather, it serves as a stress test of whether the proposed preprocessing–optimization–stacking pipeline remains effective under a different data-generating process with comparable statistical challenges.

For leakage-safe evaluation, all learning procedures (MICE imputation, SMOTE-Tomek, MI feature selection, and NSGA-II tuning) were performed within each training fold and applied only to the corresponding validation fold. The stacking configuration used was STL-Lite (XGBoost, LightGBM, CatBoost; meta-learner: XGBoost), mirroring the deployment-oriented setup in Sect. “Evaluation of STL-Lite (lightweight variant of STL-Net)”.

Under stratified 10-fold cross-validation, STL-Lite achieved 81.3% accuracy, 81.4% F1-score, and 88.6% ROC-AUC on the Pima dataset (Table 8). While lower than SGCC performance, these results indicate that the overall pipeline maintains competitive discrimination under a different application setting, supporting methodological robustness rather than dataset-specific overfitting.

**Table 8:** Performance comparison on SGCC and external Pima dataset (stratified 10-fold CV).

Dataset	Accuracy (%)	Precision (%)	Recall (%)	F1-Score (%)	ROC-AUC (%)
SGCC (STL-Net)	94.4	93.0	96.0	94.0	98.7
External (Pima, STL-Lite)	81.3	81.0	82.0	81.4	88.6

#### Performance under true class imbalance (Deployment Evaluation)

Electricity theft detection (ETD) deployments are inherently imbalanced. To evaluate performance under realistic operating conditions, an additional evaluation was conducted using the original SGCC class prevalence (positive rate ≈ 0.085). The dataset was split using stratified sampling; SMOTE-Tomek was applied to the training data only, while the test set remained untouched and imbalanced. All learning procedures were restricted to the training data to avoid leakage.

Performance was primarily evaluated using the precision–recall area under the curve (PR-AUC), which is more informative than accuracy or ROC-AUC in highly skewed classification settings. For completeness, ROC-AUC was also reported. Table 9 summarizes the results. STL-Net attains PR-AUC = 0.274 and ROC-AUC = 0.717, outperforming STL-Lite (PR-AUC = 0.253, ROC-AUC = 0.696). Both models perform above the no-skill baseline defined by the positive prevalence, confirming non-trivial discrimination of rare theft events under realistic class skew. At the default threshold (0.5), theft recall is low, reflecting an uncalibrated operating point; in deployment, thresholds would be selected using PR-based or cost-sensitive calibration to match inspection capacity and desired recall. Precision–recall and ROC curves for this true-imbalance setting are provided in the Supplementary Figures (SF12–SF13).

**Table 9:** Performance of STL-Net and STL-Lite under true class imbalance.

Model	Test Positive Rate	PR-AUC	ROC-AUC	Precision (Theft)	Recall (Theft)	F1-score (Theft)
STL-Net	0.085	0.274	0.717	0.644	0.088	0.156
STL-Lite	0.085	0.253	0.696	0.588	0.071	0.127

### Receiver operating characteristic (ROC) curve analysis

This section presents the receiver operating characteristic (ROC) curve analysis for STL-Net and its constituent base learners under the standard benchmarking protocol used for comparative analysis. This setting corresponds to the experimental configuration described in Sect. “Performance benchmarking and impact of NSGA-II Tuning”–“Comparative analysis of convergence, implementation complexity, and error budget”, in which models are trained using the full preprocessing, feature engineering, and optimization pipeline and evaluated on a held-out test partition.

This analysis is intentionally distinguished from the deployment-oriented evaluation under true class imbalance reported in Sect. “Performance under true class imbalance (deployment evaluation)”. In that setting, the test data preserve the original SGCC class prevalence (≈ 0.085), and performance is assessed primarily using precision–recall–based metrics. To avoid conflating benchmarking and deployment objectives, the corresponding ROC and precision–recall curves for the imbalanced deployment scenario are therefore reported separately in the Supplementary Figures (SF12–SF13).

**Fig. 7:**
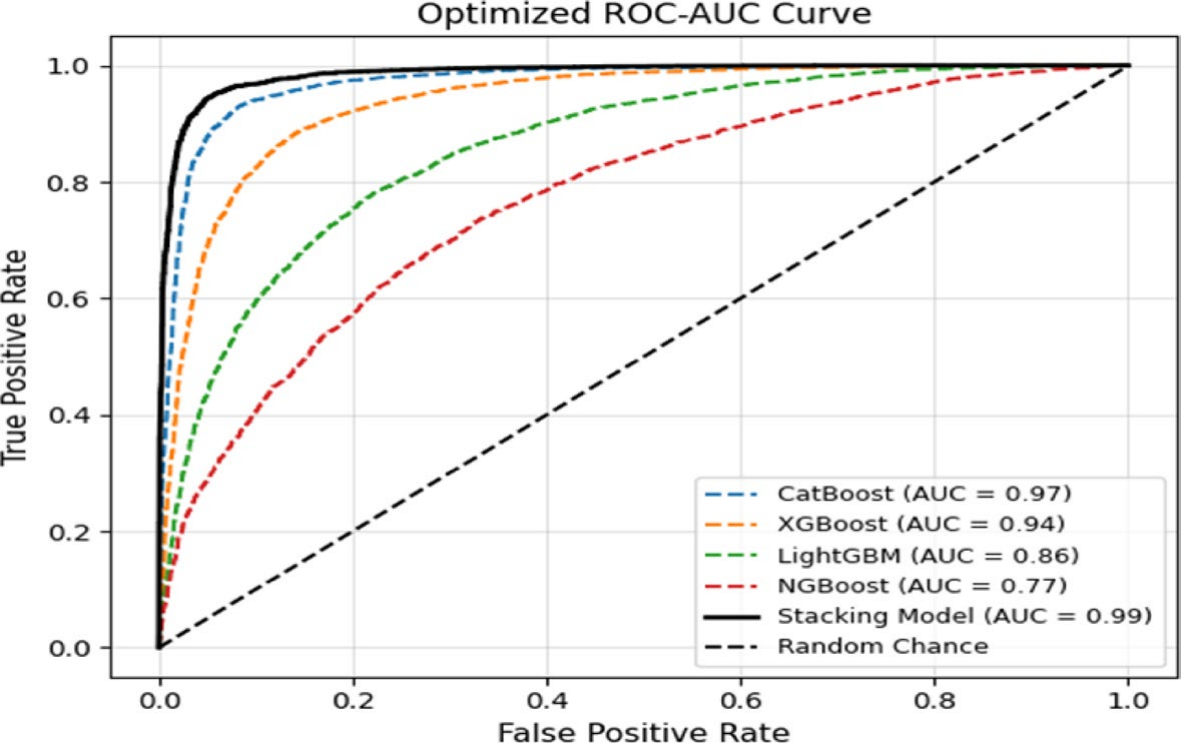
ROC curves on the held-out test set for STL-Net and base learners.

Figure 7 illustrates the ROC curves obtained on the held-out test set for STL-Net and its constituent base learners under the standard benchmarking protocol. The proposed stacking ensemble exhibits the strongest ROC curve across the full range of false-positive rates, while all individual base learners show comparatively lower separability. This visual dominance indicates that STL-Net more effectively ranks consumers by theft risk across a wide spectrum of operating thresholds.

Although the curves shown in Fig. 7 reflect performance on a single test split rather than cross-validated averages, they provide an interpretable illustration of the relative discriminative behaviour of the models under identical conditions. These observations are consistent with the cross-validated ROC-AUC results reported in Sect. Performance benchmarking and impact of NSGa-II Tuning, confirming the superior ranking capability of the proposed ensemble.

The dashed diagonal line represents a no-skill classifier (ROC-AUC = 0.50). The pronounced early rise and sustained dominance of the STL-Net curve indicate strong discrimination between fraudulent and genuine consumption patterns, particularly at low false-positive rates, which is a critical operational requirement in ETD to limit unnecessary inspections and customer disruption.

Overall, the ROC analysis confirms that STL-Net effectively integrates complementary decision boundaries learned by heterogeneous base classifiers. By resolving residual errors and inter-model conflicts at the meta-learning stage, the proposed ensemble delivers a more reliable ranking of consumers by theft risk than any individual model, reinforcing its robustness under the primary benchmarking protocol used for comparative analysis.

### Confusion matrix and error pattern analysis

**Fig. 8:**
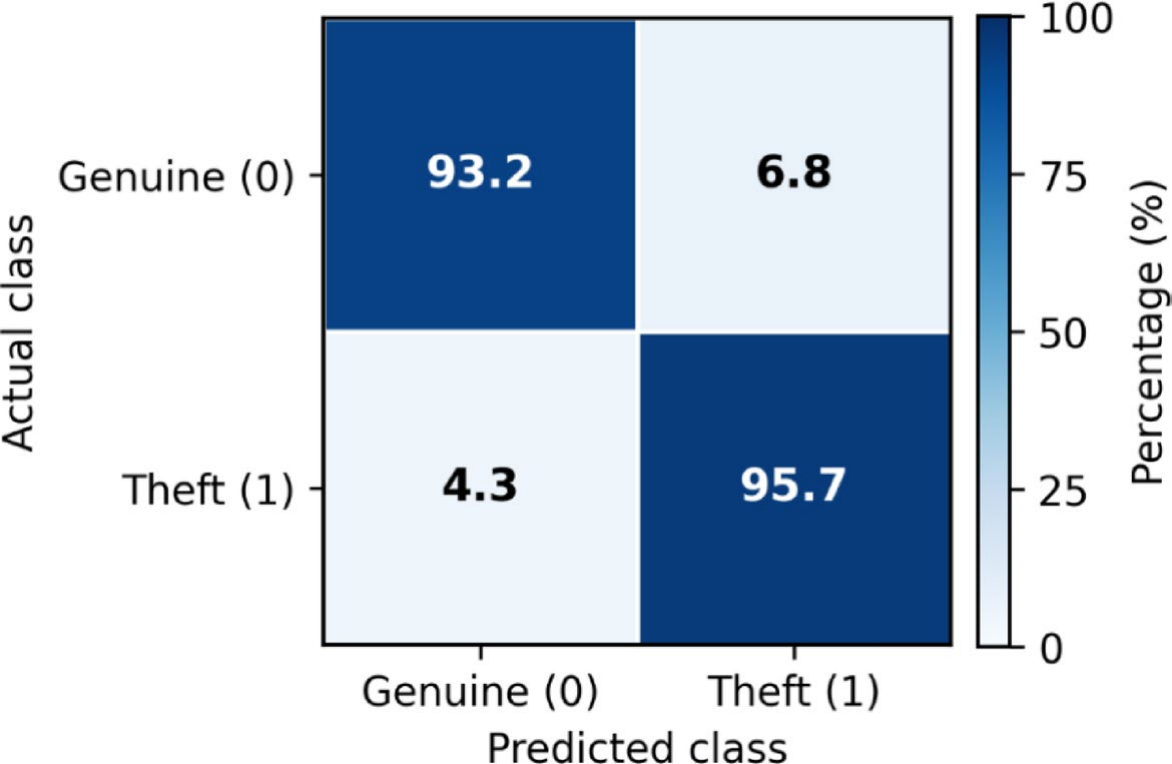
Confusion matrix for STL-Net on the test set (row-normalized, %; positive class = theft).

To further examine classification behavior at the decision threshold, a confusion matrix analysis was conducted for the proposed STL-Net model on the held-out test set under the standard benchmarking protocol. Figure 8 presents the confusion matrix in row-normalized percentage form, with the positive class corresponding to electricity theft (label = 1).

STL-Net achieves a true positive rate (TPR) of 95.7% for theft detection and a true negative rate (TNR) of 93.2% for genuine consumers. Correspondingly, the false negative rate (FNR) and false positive rate (FPR) are limited to 4.3% and 6.8%, respectively. These results indicate a well-balanced trade-off between sensitivity and specificity, consistent with the strong F1-score and agreement metrics reported in Sect. “Performance benchmarking and impact of NSGA-II Tuning”–“Statistical significance testing and performance gains”.

From an operational perspective, false negatives represent undetected theft cases and therefore pose a direct revenue risk, whereas false positives incur inspection costs and may negatively impact customer trust. To better understand these residual errors, misclassified instances were examined in conjunction with feature-level explanations derived from SHAP analysis (Sect. “Model interpretability using SHAP analysis”).

False negative cases were predominantly associated with consumers exhibiting low overall consumption combined with short-duration or weak irregularities in the most recent time windows. Such patterns often resemble seasonal variation or benign behavioral fluctuations rather than sustained theft activity, leading to reduced model confidence despite the presence of anomalies. This observation aligns with the reduced recall observed under temporal drift and noise stress tests (Sect. “Robustness and stability analysis”), where threshold calibration rather than ranking capability was identified as the primary limitation.

In contrast, false positive cases were more frequently observed among small commercial or mixed-use consumers with intermittent load profiles, particularly around billing-cycle transitions or short operational shutdowns. These users exhibit abrupt yet legitimate consumption changes that partially mimic theft-like signatures in the engineered temporal features. Importantly, these error cases were not concentrated in a specific time period but were distributed across the dataset, suggesting that they arise from structurally ambiguous consumption patterns rather than temporal bias or data leakage.

Overall, the confusion matrix analysis confirms that STL-Net maintains low misclassification rates while revealing interpretable and operationally meaningful error characteristics. These insights motivate practical enhancements such as adaptive thresholding, consumer-category-aware calibration, or the incorporation of auxiliary contextual information (e.g., tariff class or operational schedules) to further reduce false alarms and missed detections in real-world electricity theft detection deployments.

### Model interpretability using SHAP analysis

Interpretability is a critical requirement for electricity theft detection systems deployed in operational smart grid environments, where regulatory transparency, auditability, and trust in automated decisions are essential. To provide transparent explanations of model behavior, SHapley Additive exPlanations (SHAP) were employed to interpret the predictions of the proposed STL-Net framework at both global and local levels.

For tree-based base learners (XGBoost, LightGBM, and CatBoost), TreeSHAP was used to compute exact, model-consistent feature attributions with polynomial-time complexity. For the stacking ensemble output at the meta level, Kernel SHAP was applied in a model-agnostic manner to attribute the final prediction to the meta-feature matrix composed of base-learner probability outputs. To prevent information leakage, SHAP background samples were drawn exclusively from the training portion of each cross-validation fold, and explanations were evaluated on the corresponding test splits. Global feature importance was computed as the mean absolute SHAP value across all test instances, ensuring stability and comparability across folds.

**Fig. 9:**
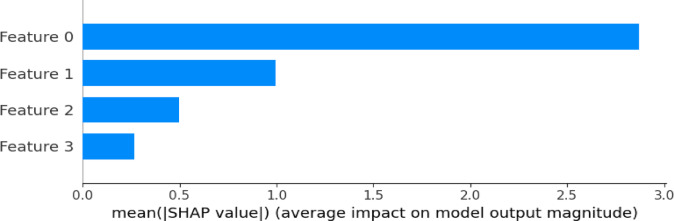
Global feature importance for STL-Net based on mean absolute SHAP values (test set). The top-ranked features correspond to PAA_49, PAA_48, PAA_47, PAA_46, matching the MI ranking in Figure SF5.

Figure 9 presents the global SHAP importance ranking for STL-Net. Feature 0 emerges as the dominant contributor to theft detection, followed by Feature 1, with additional influence from Features 2 and 3. These features correspond directly to the most recent segments of the Piecewise Aggregate Approximation (PAA) representation retained after mutual-information-based feature selection.

Specifically, Feature 0 maps to PAA_49 and Feature 1 to PAA_48, representing the most recent consumption windows before prediction. This alignment between filter-based relevance (mutual information) and model-based contribution (SHAP) confirms that the feature engineering pipeline successfully preserved the most informative temporal patterns for electricity theft detection. Importantly, the dominance of recent-window features is consistent with domain knowledge, as theft behavior often manifests through abrupt or sustained consumption suppression in the latest billing periods.

**Fig. 10:**
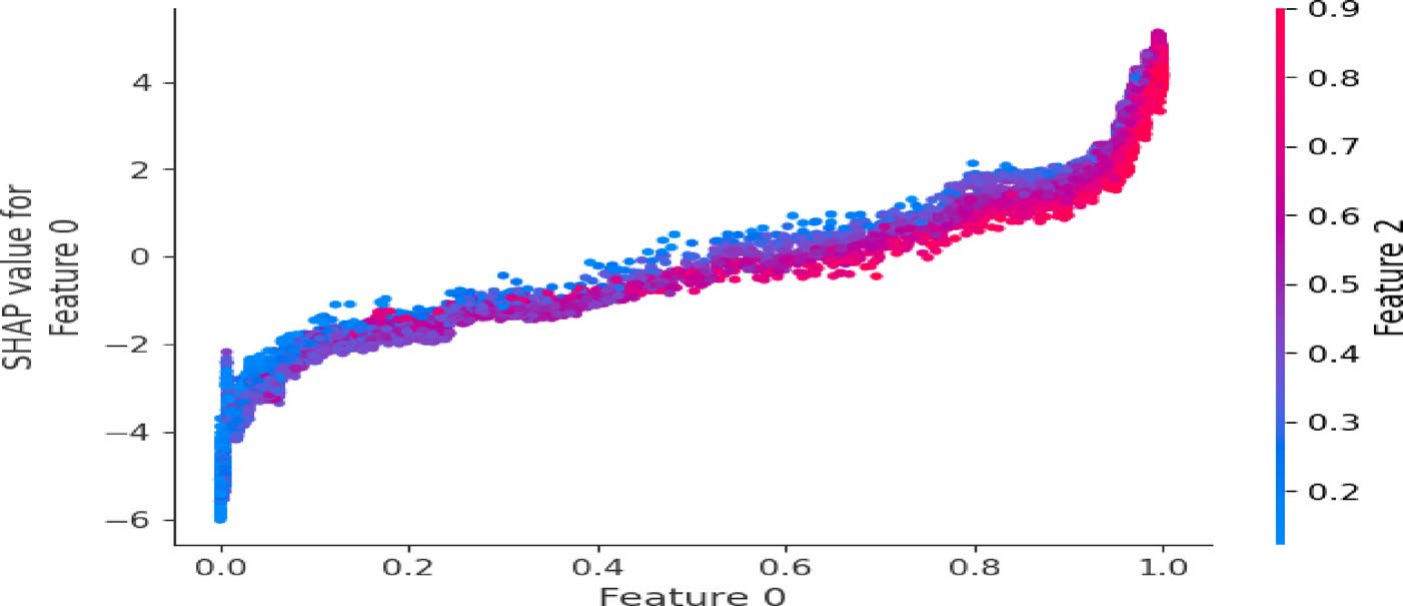
SHAP dependence plot for Feature 0 (PAA_49) colored by Feature 2.

To complement the global analysis, local SHAP explanations were examined for representative correctly classified and misclassified instances. The SHAP dependence plot shown in Fig. 10 illustrates the contribution of Feature 0 (PAA_49) as a function of its value, revealing a non-linear, monotonic increase in theft likelihood with higher feature magnitude. Coloring by Feature 2 highlights a meaningful interaction effect, whereby the influence of the most recent window is amplified when adjacent temporal segments also exhibit anomalous behavior.

This interaction pattern indicates that the model does not rely on isolated spikes but instead evaluates contextual consistency across neighboring time windows, reinforcing robustness against transient noise. Such behavior aligns with the ensemble’s improved stability under feature noise and temporal drift reported in Sect. “Robustness and stability analysis”.

For correctly detected theft cases, local SHAP force plots show strong positive contributions from recent-window PAA features, reflecting sustained consumption irregularities. In contrast, false negative cases exhibit weaker and more dispersed SHAP contributions across temporal segments, explaining the reduced prediction confidence observed in Sect. “Confusion matrix and error pattern analysis”. False positive cases are typically characterized by elevated contributions from mid-sequence features without persistent support from the most recent windows, consistent with legitimate but irregular consumption patterns (e.g., intermittent commercial usage).

Beyond explanatory value, SHAP outputs offer direct operational utility. High-risk alerts can be prioritized based on dominant contributions from recent-window features (PAA_49–PAA_48) combined with supportive evidence from adjacent segments. Moreover, local explanations can be integrated into advanced metering infrastructure (AMI) dashboards to provide transparent, auditable justifications for inspection decisions.

While SHAP values reflect statistical associations rather than causal relationships, their strong alignment with domain expectations and observed error patterns reinforces confidence in STL-Net’s decision logic. Collectively, these interpretability results demonstrate that STL-Net achieves a favorable balance between high predictive accuracy and explainable decision-making, supporting its suitability for real-world electricity theft detection deployments.

### Discussions and insights

Electricity theft detection (ETD) in operational smart grid environments is constrained by three interrelated factors: imperfect and partially adversarial data, extreme class imbalance, and deployment limits such as latency, interpretability, and inspection capacity. Although many ETD studies report high classification accuracy under controlled settings, practical utility is often reduced by assumptions of clean data, balanced class distributions, or unconstrained computational resources^13,22,25,60^. Against this backdrop, the results for STL-Net provide insights that extend beyond numerical performance gains and highlight several deployment-relevant implications.

First, the stable discrimination performance of STL-Net across cross-validation and robustness analyses indicates that explicitly modelling the accuracy–complexity trade-off can be an effective strategy for scalable ETD. In contrast to ensemble approaches that focus primarily on predictive accuracy, STL-Net incorporates Pareto-based optimization to identify configurations that balance computational efficiency with detection sensitivity. This observation aligns with prior ETD and smart grid analytics studies, which report that marginal accuracy improvements can be accompanied by disproportionate increases in inference latency and resource consumption, limiting practicality for large-scale or edge deployment^23,24,49^.

Second, the error profile observed in the confusion matrix analysis reflects a practically relevant balance between false negatives (FN) and false positives (FP). False negatives are predominantly associated with weak or short-duration irregularities that resemble benign seasonal variation, and false positives occur more frequently among small commercial or mixed-use consumers whose legitimate consumption patterns exhibit abrupt changes. Similar ambiguity-driven misclassifications have been widely reported in ETD research and are an inherent limitation of consumption-only detection approaches^16,52,60^. Notably, the FN rate remains low relative to existing benchmarks, while the FP rate is within ranges typically considered manageable in inspection-driven ETD workflows, particularly when combined with risk-based prioritization.

Third, robustness analyses under temporal drift and injected noise indicate that ranking performance (ROC-AUC) remains comparatively stable even when threshold-dependent metrics decline. Comparable behaviour has been documented in imbalanced and non-stationary detection settings^56,63^. This pattern suggests that performance degradation under drift may reflect threshold sensitivity rather than a breakdown in ranking capability. Accordingly, threshold recalibration using precision–recall or cost-sensitive criteria is commonly adopted when deploying models under evolving distributions^51,56^.

The evaluation under true class imbalance further reinforces this insight. When the original SGCC class prevalence is preserved, PR-AUC provides a more informative assessment of deployment readiness than accuracy or ROC-AUC alone, as it directly reflects the trade-off between inspection yield and coverage under scarce positive events^51,54^. The higher PR-AUC observed for STL-Net relative to STL-Lite indicates that the full ensemble retains additional discriminative capacity under severe imbalance. At the same time, the lightweight variant remains a viable option in scenarios where latency constraints are the primary consideration.

From a deployment perspective, the comparison between STL-Net and STL-Lite illustrates a clear performance–latency continuum. STL-Lite reduces inference time by approximately 40% while preserving most of the predictive performance, making it suitable for near–real-time or edge-level screening. This observation is consistent with prior findings that carefully pruned ensemble architectures can outperform deep learning models on structured smart meter data while remaining significantly more resource-efficient^22–25^. However, as latency measurements in this study are obtained on general-purpose CPU environments, further validation on embedded or utility-grade edge hardware remains an important direction for future work.

Finally, the integration of SHAP-based interpretability enables transparent attribution of detection decisions, which is increasingly recognized as a regulatory and operational requirement in smart grid analytics^92,94^. The alignment between mutual-information-based feature selection and SHAP-derived importance rankings indicates that STL-Net’s predictions are primarily influenced by recent and contextually consistent consumption patterns, rather than spurious correlations. Beyond explanatory value, such insights may inform downstream decision-support processes, including model auditing, result interpretation, and the communication of detection rationale.

Despite these encouraging results, the present study remains subject to several limitations that merit consideration. In particular, the primary evaluation is conducted on a single large-scale utility dataset, and regional deployment would require local calibration and threshold adjustment in response to evolving consumption patterns.

Overall, the discussion indicates that the primary contribution of STL-Net lies not only in improved detection accuracy but in demonstrating how Pareto-optimized ensemble learning, robustness-aware evaluation, and interpretability can be jointly operationalized for scalable electricity theft detection. While the results are encouraging, future research should extend validation across additional regional datasets, incorporate explicit cost-sensitive decision frameworks, and evaluate deployment performance on embedded edge platforms to support broader real-world applicability.

## Conclusion

This study presented STL-Net, a leakage-safe stacking ensemble framework for electricity theft detection that addresses key practical challenges in smart-meter analytics, including missing data, extreme class imbalance, high-dimensional temporal consumption patterns, and the need for transparent model behavior. By integrating hybrid data repair, temporal compression with feature selection, and Pareto-based multi-objective optimization, the proposed framework explicitly considers trade-offs between detection performance, computational efficiency, and interpretability within the evaluated experimental setup.

Across comprehensive evaluations on the SGCC dataset, STL-Net achieved consistently high discriminative performance relative to the evaluated tree-based, ensemble, and deep learning baselines, with ranking performance (ROC-AUC) exceeding 0.99 under standard conditions and remaining stable under temporal drift and noise perturbations. Statistical testing indicates that the observed performance differences are unlikely to be attributable to random variation. Under the original, highly imbalanced class distribution, precision–recall analysis further highlighted the effectiveness of STL-Net in rare-event detection scenarios.

The STL-Lite configuration demonstrates that a reduced ensemble can substantially lower inference latency while preserving most of the ranking capability of the full model, illustrating a practical performance–latency continuum for latency-sensitive or resource-constrained deployments. In addition, SHAP-based interpretability enables transparent attribution of detection outcomes, supporting model auditing and result interpretation in regulated smart grid applications.

Despite these results, the study remains subject to several limitations. The primary evaluation is conducted on a single large-scale utility dataset, and latency measurements are obtained under general-purpose CPU environments. Consequently, regional deployment would require local calibration, threshold adjustment under evolving consumption patterns, and validation on embedded or utility-grade edge hardware.

Future work will therefore focus on extending validation across diverse regional datasets, incorporating additional contextual information such as tariff structures or seasonal metadata, and exploring adaptive thresholding and online learning strategies to address long-term concept drift. Overall, this work demonstrates how Pareto-optimized ensemble learning, robustness-aware evaluation, and interpretable modeling can be jointly operationalized to support scalable electricity theft detection in modern smart-grid systems.

## Supplementary Information

Below is the link to the electronic supplementary material.

Supplementary Material 1

## Data Availability

The dataset analyzed in this study is publicly available. It was provided by the State Grid Corporation of China (SGCC) and can be accessed at: [https://github.com/henryRDlab/ElectricityTheftDetection].

## References

[1] Northeast Group. Electricity theft and non-technical losses: global markets, solutions, and vendors. (2021). https://northeast-group.com/2021/10/20/electricity-theft-non-technical-losses/

[2] Hasan, M. N., Toma, R. N., Nahid, A. A., Islam, M. M. M. & Kim, J. M. Electricity theft detection in smart grid systems: a CNN–LSTM based approach. *Energies* **12**, 3310. 10.3390/en12173310 (2019).

[3] Kumar, P. et al. Smart grid metering networks: a survey on security, privacy and open research issues. *IEEE Commun. Surv. Tutor.* **21**, 2886–2927. 10.1109/COMST.2019.2899354 (2019).

[4] Do, V. et al. Spatiotemporal distribution of power outages with climate events and social vulnerability in the USA. *Nat. Commun.* **14**, 38084. 10.1038/s41467-023-38084-6 (2023). 10.1038/s41467-023-38084-6 PMC10147900 37120649

[5] Boran, F., Mutlu, I. & Akbaş, A. A comprehensive review of advanced metering infrastructure and its applications in smart grids. *Renew. Sustain. Energy Rev.* **175**, 113183. 10.1016/j.rser.2023.113183 (2023).

[6] Ding, J. et al. Cyber threats to smart grids: review, taxonomy, potential solutions, and future directions. *Energies* **15**, 6799. 10.3390/en15186799 (2022).

[7] Abdul, R. F. & Saravanan, A. A novel data transmission model using hybrid encryption scheme for preserving data integrity. *Adv. Technol. Innov.* **10**, 15–28. 10.46604/aiti.2024.14114 (2025).

[8] Lai, J. & Heng, S. H. Secure file storage on cloud using hybrid cryptography. *J. Inf. Web Eng.* **1**, 1–18. 10.33093/jiwe.2022.1.2.1 (2022).

[9] Gržinić, M. Springer, Singapore,. Data encryption in fog computing using hybrid cryptography with integrity check. In *Proc. Int. Conf. Comput. Sci.* 627–638 (2023). 10.1007/978-981-19-6525-8_48

[10] Iqbal, M. S. et al. A critical review of technical case studies for electricity theft detection in smart grids. *Energy Convers. Manag X*. **26**, 100965. 10.1016/j.ecmx.2025.100965 (2025).

[11] Zulu, C. L. & Dzobo, O. Real-time power theft monitoring and detection system with double-connected data capture system. *Int. J. Renew. Energy Res.* **13**, 3065–3083. 10.1007/s00202-023-01825-3 (2023).

[12] Implementation of a sustainable. security architecture using radio frequency identification (RFID) technology for access control. Preprint at (2023). https://arxiv.org/abs/2304.04628

[13] Kumar, A. et al. Cost-efficient and scalable electricity theft detection in smart grids: challenges, tools, and future directions. *IEEE Access.* **11**, 80234–80256. 10.1109/ACCESS.2023.3295023 (2023).

[14] Arias-Marín, C., Barragán-Escandón, A., Toledo-Orozco, M. & Serrano-Guerrero, X. Methodological validation of machine learning models for non-technical loss detection in electric power systems. *Appl. Sci.* **15**, 3912. 10.3390/app15073912 (2025).

[15] Jiménez, D., Barrera, J. & Cancela, H. Communication network reliability under geographically correlated failures using probabilistic seismic hazard analysis. *IEEE Access.* **11**, 31341–31354. 10.1109/ACCESS.2023.3255794 (2023).

[16] Whalen, J. K. A review on game theory with smart grid security. Preprint at https://arxiv.org/abs/2304.11738 (2023). 10.48550/arxiv.2304.11738

[17] Kim, S. et al. Data-driven approaches for energy theft detection: a comprehensive review. *Energies* **17**, 3057. 10.3390/en17123057 (2024).

[18] Mu, T., Yu, Y., Feng, G., Luo, H. & Yang, H. Detecting anomalous electricity consumption with transformer and synthesized anomalies. *PeerJ Comput. Sci.* **9**, e1721. 10.7717/peerj-cs.1721 (2023). 38077596 10.7717/peerj-cs.1721 PMC10702936

[19] Shabad, P. K., Alrashide, A. & Mohammed, O. Anomaly detection in smart grids using machine learning. In *Proc. IEEE IECON* 1–8 (IEEE, 2021). 10.1109/IECON48115.2021.9589851

[20] Banik, S. et al. Anomaly detection techniques in smart grid systems: a review. In *Proc. IEEE World AI IoT Congr.* 331–337 (IEEE, 2023). 10.1109/AIIoT58121.2023.10174485

[21] Sun, Y., Sun, X., Hu, T. & Liu, H. Smart grid theft detection based on hybrid multi-time scale neural network. *Appl. Sci.* **13**, 5710. 10.3390/app13095710 (2023).

[22] Wang, Y. & Jin, S. A convolution–non-convolution parallel deep network for electricity theft detection. *Sustainability* **15**, 10127. 10.3390/su151310127 (2023).

[23] El-Toukhy, A. T. et al. Electricity theft detection using deep reinforcement learning in smart power grids. *IEEE Access.* **11**, 59558–59574. 10.1109/ACCESS.2023.3284681 (2023).

[24] Li, P. et al. Multi-model running latency optimization in an edge computing paradigm. *Sensors* **22**, 6097. 10.3390/s22166097 (2022). 36015856 10.3390/s22166097 PMC9415810

[25] Jain, T. et al. Latency-memory optimized splitting of convolution neural networks for resource-constrained edge devices. In *Proc. COMSNETS* 531–539 (IEEE, 2022). 10.1109/COMSNETS53615.2022.9668356

[26] Almalki, A. J. Unsupervised learning with hybrid models for detecting electricity theft in smart grids. *IEEE Access.* **12**, 187027–187040. 10.1109/ACCESS.2024.3498733 (2024).

[27] Hussain, S. et al. A novel unsupervised feature-based approach for electricity theft detection using robust PCA. *Int. Trans. Electr. Energy Syst.* **30**, e12572. 10.1002/2050-7038.12572 (2020).

[28] Aslam, Z. et al. A new clustering-based semi-supervised method to restrict anomalous electricity consumption. *Electr. Eng.* **106**, 6431–6448. 10.1007/s00202-024-02362-3 (2024).

[29] Gunturi, S. K. & Sarkar, D. Ensemble machine learning models for the detection of energy theft. *Electr. Power Syst. Res.* **192**, 106904. 10.1016/j.epsr.2020.106904 (2021).

[30] Fei, K., Li, Q. & Zhu, C. Non-technical losses detection using missing values’ pattern and neural architecture search. *Int. J. Electr. Power Energy Syst.* **134**, 107410. 10.1016/j.ijepes.2021.107410 (2022).

[31] Elhassan, A. et al. ILA4: overcoming missing values in machine learning datasets. *J. King Saud Univ. Comput. Inf. Sci.* **34**, 4284–4295. 10.1016/j.jksuci.2021.02.011 (2022).

[32] Tureczek, A., Nielsen, P. S. & Madsen, H. Electricity consumption clustering using smart meter data. *Energies* **11**, 859. 10.3390/en11040859 (2018).

[33] Huang, M. W. et al. Data preprocessing issues for incomplete medical datasets. *Expert Syst.* **33**, 432–438. 10.1111/exsy.12155 (2016).

[34] Patidar, S. et al. Missing data imputation for community energy demand modelling. In *Proc. IBPSA-Scotland Conf.* (2022).

[35] Zhang, Y. et al. Missing value imputation in multivariate time series with end-to-end generative adversarial networks. *Inf. Sci.* **551**, 67–82. 10.1016/j.ins.2020.11.035 (2021).

[36] Saputra, M. D. et al. Handling missing values and unusual observations using Kalman filter. *J. Phys. Conf. Ser.* **1863**, 012035. 10.1088/1742-6596/1863/1/012035 (2021).

[37] Sovilj, D. et al. Extreme learning machine for missing data using multiple imputations. *Neurocomputing* **174**, 220–231. 10.1016/j.neucom.2015.03.108 (2016).

[38] Jiang, Z. et al. Robust smart meter data analytics using smoothed ALS. *Energies* **11**, 1401. 10.3390/en11061401 (2018).

[39] Stekhoven, D. J. & Bühlmann, P. MissForest—non-parametric missing value imputation. *Bioinformatics* **28**, 112–118. 10.1093/bioinformatics/btr597 (2012). 22039212 10.1093/bioinformatics/btr597

[40] Rahman, M. G. & Islam, M. Z. Missing value imputation using fuzzy clustering-based EM. *Knowl. Inf. Syst.* **46**, 389–422. 10.1007/s10115-015-0822-y (2016).

[41] Mantuano, C. et al. Data imputation methods for intermittent renewable energy sources. *Energy Convers. Manag*. **339**, 119857. 10.1016/j.enconman.2025.119857 (2025).

[42] Zhao, J., Li, Y. & Mo, H. Handling missing data using the XGBoost-based multiple imputation by chained equations regression method. *Front. Artif. Intell.* **8**, 1553220. 10.3389/frai.2025.1553220 (2025). 40248006 10.3389/frai.2025.1553220 PMC12003350

[43] Li, L. et al. Estimation of missing values in heterogeneous traffic data. *Knowl. Based Syst.* **194**, 105592. 10.1016/j.knosys.2020.105592 (2020).

[44] Hernández, Á. et al. Detection of anomalies in daily activities using data from smart meters. *Sensors* **24**, 515. 10.3390/s24020515 (2024). 38257607 10.3390/s24020515 PMC10818482

[45] Saleh, O. A. & Cevik, M. Secure edge-based smart grid communication using lightweight authentication modeling. *Discover Comput.* **28**, 110. 10.1007/s10791-025-09643-w (2025).

[46] Molokomme, D. N. et al. Edge intelligence in smart grids: a survey. *J. Sens. Actuator Netw.* **11**, 47. 10.3390/jsan11030047 (2022).

[47] Azzalini, D. et al. An empirical evaluation of deep autoencoders for anomaly detection. *Energy Build.* **327**, 115069. 10.1016/j.enbuild.2024.115069 (2025).

[48] Jithish, J. et al. Distributed anomaly detection in smart grids using federated learning. *IEEE Access.* **11**, 7157–7179. 10.1109/ACCESS.2023.3237554 (2023).

[49] Javaid, N. et al. Adaptive synthesis to handle imbalanced big data. *J. Parallel Distrib. Comput.* **153**, 44–52. 10.1016/j.jpdc.2021.03.002 (2021).

[50] Marangu, D. K. et al. Comparative analysis of class imbalance handling techniques. *Int. J. Artif. Intell. Appl.* **15**, 13–27. 10.5121/ijaia.2024.15602 (2024).

[51] Bunkhumpornpat, C., Sinapiromsaran, K. & Lursinsap, C. DBSMOTE: density-based synthetic minority over-sampling technique. *Appl. Intell.* **36**, 664–684. 10.1007/s10489-011-0287-y (2012).

[52] Koziarski, M. Radial-based undersampling for imbalanced data classification. *Pattern Recognit.* **102**, 107262. 10.1016/j.patcog.2020.107262 (2020).

[53] Buda, M., Maki, A. & Mazurowski, M. A. Systematic study of the class imbalance problem in CNNs. *Neural Netw.* **106**, 249–259. 10.1016/j.neunet.2018.07.011 (2018). 30092410 10.1016/j.neunet.2018.07.011

[54] Sáez, J. A., Krawczyk, B. & Woźniak, M. Oversampling strategies in multi-class imbalanced datasets. *Pattern Recognit.* **57**, 164–178. 10.1016/j.patcog.2016.03.012 (2016).

[55] Adil, M. et al. LSTM and bat-based RUSBoost approach for electricity theft detection. *Appl. Sci.* **10**, 4378. 10.3390/app10124378 (2020).

[56] Naeem, A. et al. Deep fractal network with light gradient boosting approach. *Heliyon* **9**, e18928. 10.1016/j.heliyon.2023.e18928 (2023). 37681137 10.1016/j.heliyon.2023.e18928 PMC10480595

[57] Wu, J. C., Lu, S., Fuh, C. S. & Liu, T. L. One-class anomaly detection via novelty normalization. *Comput. Vis. Image Underst.* **210**, 103226. 10.1016/j.cviu.2021.103226 (2021).

[58] Buzau, M. M. et al. Detection of non-technical losses using smart meter data and supervised learning. *IEEE Trans. Smart Grid*. **10**, 2661–2670. 10.1109/TSG.2018.2807925 (2019).

[59] Lin, M. et al. Detection of ionospheric scintillation using XGBoost improved by SMOTE-ENN. *Remote Sens.* **13**, 2577. 10.3390/rs13132577 (2021).

[60] Galar, M., Fernández, A., Barrenechea, E. & Herrera, F. EUSBoost: enhancing ensembles for highly imbalanced datasets. *Pattern Recognit.* **46**, 3460–3471. 10.1016/j.patcog.2013.05.006 (2013).

[61] Azeem, A. et al. Mitigating concept drift challenges in evolving smart grids. *Energy Rep.* **13**, 1369–1383. 10.1016/j.egyr.2024.12.078 (2025).

[62] Tripathi, A. K., Pandey, A. C. & Sharma, N. Hybrid adaptive sampling and pipeline machine learning for electricity theft detection. *Multimed Tools Appl.* **83**, 54521–54544. 10.1007/s11042-023-17730-7 (2024).

[63] Liu, Z. et al. Electricity theft detection through contrastive learning. *J. Wirel. Commun. Netw.* **2023, **54 (2023). 10.1186/s13638-023-02258-z

[64] Gong, X. & Ma, L. Smart meter outlier detection using PCA dimensionality reduction and K-means clustering. In *Proc. 4th Int. Conf. Robotics, Automation and Intelligent Control (ICRAIC)* 444–447 (IEEE, 2024). 10.1109/ICRAIC65937.2024.00084

[65] Shamim, G. & Rihan, M. Exploratory data analytics and PCA-based dimensionality reduction for smart meter data clustering. *IETE J. Res.* **70**, 4159–4168. 10.1080/03772063.2023.2218317 (2024).

[66] Razavi, R. et al. Practical feature-engineering framework for electricity theft detection. *Appl. Energy*. **238**, 481–494. 10.1016/j.apenergy.2019.01.076 (2019).

[67] Dong, G. G. & Liu, H. *Feature Engineering for Machine Learning and Data Analytics* (CRC, 2018). 10.1201/9781315181080

[68] Ibrahim, M. S., Dong, W. & Yang, Q. Machine learning driven smart electric power systems. *Appl. Energy*. **272**, 115237. 10.1016/j.apenergy.2020.115237 (2020).

[69] Saeed, M. S. et al. Boosted C5.0 decision-tree-based classification for non-technical loss detection. *Energies* **13**, 3242. 10.3390/en13123242 (2020).

[70] Punmiya, R. & Choe, S. Energy theft detection using gradient boosting theft detector. *IEEE Trans. Smart Grid*. **10**, 2326–2329. 10.1109/TSG.2019.2892595 (2019).

[71] Lee, L. C. & Jemain, A. A. PCA application strategy in high dimensional forensic data. *Microchem J.* **169**, 106608. 10.1016/j.microc.2021.106608 (2021).

[72] Gul, H. et al. Detection of non-technical losses using SOSTLink and bidirectional GRU. *Appl. Sci.* **10**, 3151. 10.3390/app10093151 (2020).

[73] Davila Delgado, J. M. & Oyedele, L. Deep learning with small datasets using autoencoders. *Appl. Soft Comput.* **112**, 107836. 10.1016/j.asoc.2021.107836 (2021).

[74] Khan, Z. A. et al. Electricity theft detection using supervised learning techniques. *Sustainability* **12**, 8023. 10.3390/su12198023 (2020).

[75] Munawar, S. et al. Electricity theft detection using a hybrid BiGRU–BiLSTM model. *Sensors* **22**, 7818. 10.3390/s22207818 (2022). 36298168 10.3390/s22207818 PMC9609745

[76] Zheng, K. et al. A novel combined data-driven approach for electricity theft detection. *IEEE Trans. Ind. Inf.* **15**, 1809–1819. 10.1109/TII.2018.2873814 (2019).

[77] Elshennawy, N. M., Ibrahim, D. M. & Gab Allah, A. M. Efficient electricity theft detection based on deep learning. *Sci. Rep.* **15**, 12866. 10.1038/s41598-025-93140-z (2025). 40234553 10.1038/s41598-025-93140-z PMC12000372

[78] Mbey, C. F. et al. Electricity theft detection in a smart grid using hybrid deep learning-based data analysis technique. *J. Electr. Comput. Eng.* **2024** (6225510). 10.1155/2024/6225510 (2024).

[79] Iftikhar, H. et al. Electricity theft detection in smart grid using machine learning. *Front. Energy Res.* **12**, 1383090. 10.3389/fenrg.2024.1383090 (2024).

[80] He, Y. et al. Electricity theft detection based on minimal gated memory network. *Sustain. Energy Grids Netw.* **39**, 101415. 10.1016/j.segan.2024.101415 (2024).

[81] Abro, S. A. et al. Machine learning-based electricity theft detection using support vector machines. *Int. J. Electr. Comput. Eng.* **14**, 1240–1250. 10.11591/ijece.v14i2.pp1240-1250 (2024).

[82] Cai, Q., Li, P. & Wang, R. Electricity theft detection based on hybrid random forest and weighted SVDD. *Int. J. Electr. Power Energy Syst.* **153**, 109283. 10.1016/j.ijepes.2023.109283 (2023).

[83] Yang, X. et al. TLEL: a two-layer ensemble learning approach. *Inf. Softw. Technol.* **87**, 206–220. 10.1016/j.infsof.2017.03.007 (2017).

[84] Mohammed, A. R. & Idris, R. M. Enhancing diabetes mellitus onset prediction through ensemble learning. *J. Stat. Model. Anal.* **6**, 11–28. 10.22452/josma.vol6no2.2 (2024).

[85] Al-Mayyahi, S. et al. Machine learning techniques for solar power output predicting. *Int. J. Smart Grid*. **8**, 98–107. 10.20508/ijsmartgrid.v8i2.341.g356 (2024).

[86] Hussain, S. et al. Electric theft detection in advanced metering infrastructure using Jaya-optimized boosting classifier. *IET Gener Transm Distrib.* **16**, 1257–1275. 10.1049/gtd2.12386 (2022).

[87] Zhang, Y., Liu, J. & Shen, W. Review of ensemble learning algorithms in remote sensing. *Appl. Sci.* **12**, 8654. 10.3390/app12178654 (2022).

[88] Satyapal, K. S. & Patil, A. Enhancing electricity theft detection using XGBoost with Optuna optimization. In *Proc. IEEE ISGT Asia* 1–6 (IEEE, 2024). 10.1109/ISGTAsia61245.2024.10876295

[89] Rahaman, M. A. & Idris, R. M. Enhancing energy fraud detection with ensemble learning techniques. In *Proc. IEEE APEE* 159–164 (IEEE, 2024). 10.1109/APEE60256.2024.10790893

[90] Hussain, S. et al. Feature-engineered CatBoost-based supervised machine learning framework. *Energy Rep.* **7**, 4425–4436. 10.1016/j.egyr.2021.07.008 (2021).

[91] Ullah, A. et al. Hybrid deep neural network for electricity theft detection. *Wirel. Commun. Mob. Comput.* **2021** (9933111). 10.1155/2021/9933111 (2021).

[92] Hussain, S. et al. Feature-engineered NGBoost machine-learning framework for fraud detection. *Sensors* **21**, 8423. 10.3390/s21248423 (2021). 34960516 10.3390/s21248423 PMC8704372

[93] Garciarena, U. & Santana, R. Interaction between missing data types, imputation methods, and supervised classifiers. *Expert Syst. Appl.* **89**, 52–65. 10.1016/j.eswa.2017.07.026 (2017).

[94] Alwateer, M. et al. Missing data imputation: a comprehensive review. *J. Comput. Commun.* **12**, 53–75. 10.4236/jcc.2024.1211004 (2024).

[95] Blank, J. & Deb, K. Pymoo: multi-objective optimization in python. *IEEE Access.* **8**, 89497–89509. 10.1109/ACCESS.2020.2990567 (2020).

[96] National Institute of Diabetes and Digestive and Kidney Diseases. PIMA Indians diabetes database. Kaggle (2016). https://www.kaggle.com/datasets/uciml/pima-indians-diabetes-database

