# Supplementary material for: A stacking ensemble with Pareto optimization for scalable electricity theft detection via hybrid data repair and lightweight deployment
Source: Sci Rep. 2026 Mar 23;16:14548. doi: 10.1038/s41598-026-39693-z (PMC13153380; doi:10.1038/s41598-026-39693-z)
Supplement: Supplementary file 1 — Supplementary Material 1 [file 41598_2026_39693_MOESM1_ESM.docx]

A Stacking Ensemble with Pareto Optimization for Scalable Electricity Theft Detection via Hybrid Data Repair and Lightweight Deployment

**Mohammed Ateequr Rahaman^1^*, Rasyidah Mohamad Idris^1^**

^1^Faculty of Electrical Engineering, Universiti Teknologi Malaysia, Johor Bahru, Johor, Malaysia

*Corresponding author: [mohammedrahaman@graduate.utm.my](mailto:mohammedrahaman@graduate.utm.my)

## Abbreviations & Acronyms

AdaBoost — Adaptive Boosting

AMI — Advanced Metering Infrastructure

ARIMA — AutoRegressive Integrated Moving Average

AUC — Area Under the Curve

CatBoost — Categorical Boosting

CNN — Convolutional Neural Network

CNN-LSTM — Convolutional Neural Network with Long Short-Term Memory

CV — Cross-Validation

DL — Deep Learning

DT — Decision Tree

ETC — Extra Trees Classifier

ETD — Electricity Theft Detection

F1-score — Harmonic Mean of Precision and Recall

GBM — Gradient Boosting Machine

KNN — k-Nearest Neighbors

LDA — Linear Discriminant Analysis

LightGBM — Light Gradient Boosting Machine

LR — Logistic Regression

LSTM — Long Short-Term Memory

MCC — Matthews Correlation Coefficient

MI — Mutual Information

MICE — Multiple Imputation by Chained Equations

ML — Machine Learning

NGBoost — Natural Gradient Boosting

NSGA-II — Non-dominated Sorting Genetic Algorithm II

NTL — Non-Technical Losses

PAA — Piecewise Aggregate Approximation

PCA — Principal Component Analysis

QDA — Quadratic Discriminant Analysis

RF — Random Forest

RNN — Recurrent Neural Network

ROC — Receiver Operating Characteristic

ROC-AUC — Area Under the Receiver Operating Characteristic Curve

SHAP — SHapley Additive exPlanations

SMOTE — Synthetic Minority Oversampling Technique

SMOTE-Tomek — Synthetic Minority Oversampling Technique with Tomek Links

STL-Lite — Lightweight Variant of Scalable Trustworthy Lightweight Network

STL-Net — Scalable Trustworthy Lightweight Network

SVM — Support Vector Machine

t-SNE — t-distributed Stochastic Neighbor Embedding

XGBoost — eXtreme Gradient Boosting

## Supplementary Notes

## Supplementary Notes (SN1)

Hybrid Imputation Pipeline: Mathematical Formulation, Parameter Settings, and Sensitivity Analysis

**Overview of the Hybrid Imputation Strategy**

This note provides detailed mathematical formulations, parameter configurations, and robustness analyses for the hybrid imputation pipeline employed in the STL-Net framework. The pipeline was designed to address heterogeneous missingness patterns in large-scale smart meter datasets while preserving temporal consistency, multivariate dependencies, and downstream detection performance.

The imputation process follows a sequential, sparsity-aware design, in which different techniques are applied based on feature-level missingness severity, followed by a final statistical stabilization step. This staged approach mitigates the limitations of single-method imputation under adversarial or non-uniform data loss.

**Missingness Characterisation and Threshold Selection**

Let $X\in\mathbb{R}^{n\times p}$denote the consumption matrix, where $n$is the number of consumers and $p$ =1035 represents daily consumption attributes. Feature-level missingness was quantified as the proportion of missing entries per column.

An empirical coverage analysis across all 42,372 consumers revealed a distinct transition in data availability around 30% missingness, separating highly sparse features from moderately sparse ones. Sensitivity analysis over missingness thresholds ranging from 10% to 50% demonstrated that a 30% cutoff provides an optimal balance between retained consumers and detection performance, as detailed in Section SN1.8.

Accordingly:

- Features with >30% missing values were treated as highly sparse.
- Features with ≤30% missing values were treated as moderately sparse.

This resulted in:

- 584 highly sparse features processed via MICE, and
- 451 moderately sparse features processed via KNN imputation.

**Stage I: Multiple Imputation by Chained Equations (MICE)**

For highly sparse features, Multiple Imputation by Chained Equations (MICE) was applied to recover large contiguous gaps while preserving multivariate dependencies.

Let $X\in\mathbb{R}^{n\times p}$ denote the feature set, where $X_{j}$ denotes the *j*-th feature containing missing values, and $X_{-j}$ represents all remaining features shown in equation (1).

At iteration *t*+1, missing values in $X_{j}$ are updated as:

$$\begin{aligned} X_{j}^{\left( t+1 \right)}= f_{j} \left( X_{-j}^{t} \right)+\varepsilon\#(1) \end{aligned}$$

where:

- $f_{j} \left( \cdot\right)$is the regression model fitted to observed entries,
- ε is a stochastic residual term.

The procedure iterates over all incomplete features until convergence, which is effective under missing-at-random assumptions and serves as an initial reconstruction step before robustness-oriented refinement stages. This stage effectively reconstructs large missing segments while maintaining cross-feature correlations.

**Stage II: k-Nearest Neighbours (KNN) Imputation**

For moderately sparse features, KNN imputation was applied to exploit local similarity among consumption profiles shown in equation (2).

A missing entry $X_{ij}$is estimated as:

$$\begin{aligned} X_{ij}= \frac{1}{k} \sum_{i^{'} \in N\left( i \right)} X_{i^{'}j} \#(2) \end{aligned}$$

where *N(i)* denotes the index set of the *k* nearest neighbors of instance *i*, identified using Euclidean distance (equation (3)):

$$\begin{aligned} d\left( i, i^{'} \right)= \sqrt{\sum_{n=1}^{p} \left( X_{in}- X_{i^{'}n} \right)^{2}} \#(3 \end{aligned})$$

The value of *k* in equation (3) was selected based on established practice in high-dimensional imputation literature and preliminary validation experiments, balancing local fidelity and smoothing stability. KNN complements MICE by refining features with smaller missing fractions at lower computational cost.

**Stage III: XGBoost-Based Refinement**

Despite prior imputation, residual inconsistencies such as negative or implausible consumption values may persist. To address this, XGBoost regression was employed as a refinement stage as shown in equation (4).

The objective function optimized by XGBoost is:

$$\begin{aligned} L\left( \theta\right)= \sum_{i=1}^{n} \mathcal{l}\left( y_{i}, \hat{y}_{i} \right)+ \sum_{k=1}^{T} \Omega\left( f_{k} \right) \#(4) \end{aligned}$$

with regularization shown in equation (5):

$\begin{aligned} \Omega\left( f_{k} \right)= \gamma T+ \frac{1}{2} \lambda{\mid\mid w\mid\mid}^{2} \#(5) \end{aligned}$

Here:

- $l(\cdot)$is the loss function (mean squared error),
- $T$is the number of leaves,
- $\gamma$and $\lambda$control model complexity.

Iterative application reduced anomalous values from 263,614 → 2,120 → 271, ensuring high-fidelity reconstruction of nonlinear temporal patterns.

**Stage IV: Median Adjustment and Distributional Stabilization**

Zeros may increase temporarily after intermediate imputation steps due to conservative replacement under sparse regimes; final median adjustment removes implausible zeros while preserving legitimate low-consumption readings. This step enforces physical plausibility while avoiding distortion of consumption distributions. The progression for a sample consumer profile is shown in Figure SF5, with aggregated results in Table ST3.

**Logarithmic Transformation**

To mitigate right-skewness from high-usage consumers, a logarithmic transformation was applied post-imputation. As shown in Table ST4, mean skewness across representative dates decreased from 85.67 to 0.48, substantially improving distributional symmetry and model generalization.

**Sensitivity Analysis of Missingness Threshold**

A sensitivity analysis was conducted by varying the missingness threshold from 10% to 50%, evaluating retained consumers and downstream detection metrics. Results (Supplementary Table ST2 and Supplementary Figure SF4) show that:

- Lower thresholds (≤20%) discard a substantial portion of consumers and degrade recall.
- Higher thresholds (≥40%) increase noise propagation, reducing F1-score stability.
- A 30% threshold yields the most favourable trade-off, achieving balanced retention, ROC-AUC, and F1-score.

This empirically validates the robustness of the selected threshold and supports its use in the main framework.

**Summary**

Overall, the proposed hybrid imputation strategy restores data completeness while preserving structural, temporal, and statistical properties critical for electricity theft detection. The staged design, validated through sensitivity analysis, ensures robustness against adversarial missingness and provides a reliable foundation for downstream learning in large-scale smart grid environments.

## Supplementary Note (SN2)

Class Balancing and Feature Engineering Details

This supplementary note provides detailed mathematical formulations, parameter settings, and diagnostic analyses supporting the class balancing and feature engineering stages of the proposed STL-Net framework. These details are omitted from the main manuscript for conciseness but are included here to ensure transparency, reproducibility, and methodological rigor.

**Adaptive Class Rebalancing Using SMOTE–Tomek**

The SGCC dataset exhibits severe class imbalance, with fraudulent consumers constituting approximately 8.54% of the total population. To mitigate majority-class bias while preserving decision boundary integrity, a hybrid Synthetic Minority Oversampling Technique with Tomek Links (SMOTE–Tomek) strategy was employed.

As shown in equation (6) SMOTE generates synthetic minority samples by interpolating between each minority instance and its *k* nearest minority neighbors in feature space. Formally, a synthetic instance $x_{\text{new}}$is generated as:

$$\begin{aligned} x_{\text{new}}=x_{i}+\lambda\left( x_{nn}-x_{i} \right),\lambda\in\left( 0,1 \right)\#(6) \end{aligned}$$

where $x_{i}$is a minority instance and $x_{nn}$is one of its *k* nearest neighbors.

While oversampling improves minority recall, it may introduce overlapping samples near class boundaries. Tomek Links are therefore applied to identify and remove ambiguous majority–minority pairs that share minimal inter-class distance, thereby enhancing class separability and reducing noise amplification.

All resampling operations were applied exclusively within training folds during cross-validation to prevent information leakage. The test partition retained the original class distribution and was not subjected to any resampling. Supplementary Figure (SF6) illustrates the class distribution before and after SMOTE–Tomek rebalancing.

**Visualization of Class Separability**

To qualitatively assess the impact of SMOTE–Tomek on class structure, t-distributed stochastic neighbor embedding (t-SNE) was applied to the feature space before and after rebalancing.

The visualization demonstrates that SMOTE–Tomek improves the spatial separation between genuine and fraudulent instances by reducing overlap regions that typically contribute to false negatives. Supplementary Figure (SF7) presents the t-SNE projections before and after class balancing.

**Temporal Compression via Piecewise Aggregate Approximation**

Long-horizon daily consumption profiles consist of 1,035-time steps per consumer, resulting in high-dimensional input spaces. To reduce dimensionality while preserving dominant temporal trends, Piecewise Aggregate Approximation (PAA) was applied shown in equation (7). PAA partitions each standardized time series into equal-length segments and replaces each segment with its mean value:

$$\begin{aligned} {PAA}_{j}= \frac{1}{N} \sum_{i =\left( j-1 \right)N+1}^{j.N} x_{i} \#(7) \end{aligned}$$

where $j$ denotes the segment index, $N$is the segment length, and $x_{i}$represents the original consumption value equation (7). In this study, the daily profiles were compressed into 50 aggregated segments (approximately 20 days per segment), reducing dimensionality by over 95% while retaining interpretable consumption patterns relevant to theft detection. Supplementary Figure (SF8) compares the original standardized time series with their PAA representations.

**Feature Selection Using Mutual Information**

Following temporal compression, Mutual Information (MI) was used to quantify the dependency between each PAA-derived feature and the target label. In equation (8) MI captures both linear and nonlinear relationships and is defined as:

$$\begin{aligned} MI\left( X,Y \right)= \sum_{x\in X} \sum_{y\in Y} P\left( x,y \right) . \log\left( \frac{P\left( x,y \right)}{P\left( x \right). P\left( y \right)} \right) \#(8) \end{aligned}$$

where $X$denotes a feature and $Y$ the class label.

MI scores were computed for all PAA features and ranked in descending order. Inspection of the MI ranking curve indicated a marked drop in marginal relevance beyond the top-ranked features; therefore, the top 30 were retained for downstream learning. These cutoff balances interpretability and generalization while avoiding overfitting from weakly informative dimensions. Supplementary Figure (SF9) shows the MI ranking curve used to guide feature selection.

**Summary and Reproducibility**

The combination of SMOTE–Tomek rebalancing, PAA-based temporal compression, and MI-driven feature selection yields a compact, discriminative, and well-conditioned feature space. These operations collectively enhance detection sensitivity under severe class imbalance while maintaining computational efficiency and interpretability. All hyperparameters and procedures were applied consistently across experiments, with tuning and resampling restricted to training folds to prevent leakage.

## Supplementary Note (SN3)

**Robustness under Feature Noise**

This supplementary note provides a detailed analysis of the robustness of the proposed STL-Net framework under feature-level noise perturbations. Feature noise robustness is an important consideration in electricity theft detection (ETD), as smart-meter data are frequently affected by measurement errors, communication noise, and preprocessing artefacts that may distort consumption values without altering underlying behavioral patterns.

**Noise Injection Protocol**

To evaluate robustness, controlled stochastic noise was injected into the input feature space of the test data while keeping the training data unchanged. Noise was applied to the compressed feature representation obtained after Piecewise Aggregate Approximation (PAA) and mutual information–based feature selection. Perturbations were modeled as zero-mean Gaussian noise with increasing variance levels, simulating progressively degraded measurement quality. All experiments were conducted using the same trained models to isolate the effect of noise on inference performance.

**Evaluation Metrics**

Model performance under noise was assessed using both ranking-based and threshold-dependent metrics, including ROC–AUC, precision, recall, and F1-score. This dual evaluation enables differentiation between degradation in discriminatory capability and sensitivity to decision thresholds under perturbed inputs.

**Results and Observations**

As reported in **Supplementary Tables ST10–ST12**, STL-Net maintained stable ranking performance across all evaluated noise levels, with ROC–AUC exhibiting only minor variation relative to the clean test condition. This indicates that the ensemble retains its ability to correctly rank consumers by theft likelihood even when feature values are partially corrupted. In contrast, threshold-dependent metrics such as accuracy and F1-score showed greater sensitivity to increasing noise, reflecting shifts in the operating point rather than a loss of underlying discriminatory information.

The observed behavior is consistent with the temporal drift experiments reported in the main manuscript, where ranking stability was preserved while threshold-dependent performance varied more noticeably. These results suggest that performance degradation under feature noise is primarily associated with threshold sensitivity rather than structural instability of the learned model.

**Comparison with STL-Lite**

The lightweight STL-Lite variant exhibited similar qualitative trends under noise perturbation, though with slightly increased variability in threshold-dependent metrics compared to the full STL-Net ensemble. This behavior reflects the reduced model redundancy in the lightweight configuration and is consistent with the performance–complexity trade-off discussed in the main text. Importantly, ranking performance for STL-Lite remained stable across noise levels, indicating that the lightweight design does not compromise core discriminatory capability under moderate feature corruption.

**Implications**

Overall, the noise robustness analysis confirms that STL-Net and its lightweight variant demonstrate resilience to feature-level perturbations commonly encountered in real-world smart-meter data. The preservation of ranking performance under noise supports the suitability of the proposed framework for deployment scenarios where measurement uncertainty is unavoidable, provided that appropriate threshold calibration strategies are applied.

## Supplementary Note (SN4)

**Real-World Deployment Considerations**

This supplementary note outlines practical considerations for deploying the proposed STL-Net framework within advanced metering infrastructure (AMI) environments. The intent is to illustrate feasible implementation patterns and operational aspects that may be relevant when translating the proposed methods from experimental evaluation to applied settings. These considerations are not part of the core methodological contributions and are therefore provided for completeness.

**Model Configurations and Latency Characteristics**

Two deployment configurations were evaluated in the main manuscript: the full STL-Net ensemble and the lightweight STL-Lite variant. Under CPU-only inference, STL-Net achieved higher overall detection performance with an average inference latency of approximately 6.0 ms per record, whereas STL-Lite reduced inference latency to approximately 3.9 ms per record by removing higher-complexity ensemble components. These configurations illustrate a performance–latency continuum that may be relevant for deployments with differing computational constraints.

**Illustrative Infrastructure Patterns**

In applied settings, model inference may be implemented using either decentralized or centralized architectures. One possible configuration involves containerized inference services located close to data concentrators to minimize latency, while an alternative configuration places inference within centralized microservices accessed via message-based streaming systems. In both cases, lightweight container images exposing standard REST or gRPC interfaces can support synchronous or batched scoring, depending on throughput requirements.

**Training and Inference Separation**

The experimental results indicate that inference for both STL-Net and STL-Lite is computationally efficient on general-purpose CPU hardware, whereas training and multi-objective optimization represent more computationally intensive processes. In practice, this separation enables training and hyperparameter optimization to be conducted offline, with only finalized model artefacts deployed for inference. The reported training times therefore reflect development-stage costs rather than operational inference requirements.

**Threshold Selection and Calibration Considerations**

As discussed in the robustness analysis, ranking performance remains stable under temporal drift and feature perturbations, while threshold-dependent metrics exhibit greater sensitivity. This behavior highlights the role of decision-threshold selection in applied ETD scenarios. In practice, probability calibration and threshold adjustment may be performed using recent validation data to align model outputs with operational objectives, such as inspection capacity or acceptable false-positive rates. The specific calibration strategy is deployment-dependent and beyond the scope of this study.

**Monitoring and Model Maintenance**

Operational deployments may benefit from ongoing monitoring of input data characteristics, score distributions, and error profiles to identify shifts relative to training conditions. Such monitoring can support informed decisions regarding recalibration or retraining when consumption patterns evolve. The experimental framework presented in this study is compatible with periodic model updates using newly observed data, provided that leakage-safe training protocols are preserved.

**Explainability in Applied Settings**

The SHAP-based interpretability approach used in the main manuscript is primarily evaluated offline. In applied settings, similar attribution summaries may be generated to support post-hoc analysis of model outputs, enabling reviewers or analysts to understand which temporal features contributed most strongly to individual predictions. This capability supports transparency and auditability without altering the underlying detection process.

**Data Governance and Regional Adaptation**

Deployment of ETD models in operational environments typically requires adherence to data governance, privacy, and security policies that vary across jurisdictions. While the proposed framework was validated using SGCC data, regional adaptation may involve recalibration to local consumption profiles, seasonal characteristics, and class prevalence. Such adaptation can be performed using the same leakage-safe training and validation procedures described in the main manuscript.

**Summary**

This supplementary note provides contextual guidance on how the proposed STL-Net framework may be integrated into AMI-scale workflows. The examples presented illustrate feasible deployment patterns and maintenance considerations but do not constitute prescriptive operational recommendations. The primary scientific contributions of this work remain the methodological framework, robustness analysis, and empirical evaluation presented in the main manuscript.

## Supplementary Tables

## Supplementary Table (ST1)

**Table ST1.** Mapping of ETD Challenges, Limitations of Existing Methods, and STL-Net Solutions

| **Challenge** | **Common approaches** | **Limitations** | **STL-Net Solution** |
| --- | --- | --- | --- |
| Missing Data | Mean, Median, Linear Interpolation, ARIMA | Information loss, biased estimates | Hybrid imputation with MICE, KNN, and XGBoost |
| Class Imbalance | Oversampling, Undersampling, SMOTE | Overfitting, poor generalization | SMOTE-Tomek to enhance minority representation and reduce noise |
| High Dimensionality | PCA, Kernel PCA | Loss of interpretability, noise sensitivity | PAA for interpretability-preserving compression of temporal profiles |
| Feature Relevance | Statistical descriptors | Inability to capture non-linear or multivariate relevance | Mutual Information for robust, relevance-based feature selection |
| Model Selection & Optimization | SVM, RF, XGBoost | Overfitting, imbalance sensitivity, and limited diversity | Stacking ensemble with NGBoost, CatBoost, LightGBM, and XGBoost, optimized via NSGA-II |
| Interpretability | Decision Trees, Black-Box Models | Poor transparency, low stakeholder trust | SHAP integration for feature-level transparency and regulatory explainability |
| Model Evaluation | Accuracy, Precision, Recall | Incomplete performance assessment | Multi-metric evaluation using ROC-AUC, F1-score, Cohen’s kappa, and MCC |

## Supplementary Table (ST2)

**Table ST2.** Missingness threshold sensitivity analysis (retained consumers, class ratio, ROC-AUC, F1-score, accuracy, precision, recall)

| **missing_threshold_%** | **retained_consumers** | **pos_rate** | **roc_auc** | **f1** | **accuracy** | **Preci-sion** | **recall** |
| --- | --- | --- | --- | --- | --- | --- | --- |
| 10 | 20997 | 0.065 | 0.769 | 0.088 | 0.936 | 0.619 | 0.047 |
| 20 | 25594 | 0.068 | 0.756 | 0.101 | 0.934 | 0.704 | 0.054 |
| 30 | 27994 | 0.074 | 0.788 | 0.158 | 0.930 | 0.673 | 0.090 |
| 40 | 29695 | 0.076 | 0.792 | 0.139 | 0.925 | 0.537 | 0.080 |
| 50 | 31188 | 0.077 | 0.803 | 0.123 | 0.924 | 0.589 | 0.068 |

## Supplementary Table (ST3)

**Table ST3.** Progressive refinement of missing data through hybrid imputation

| **Dataset** | **Total Zeros** | **Total Negative Values** | **Total Missing Values** |
| --- | --- | --- | --- |
| Raw Dataset | 5,788,603 | 0 | 11,233,528 |
| Hybrid Imputed (MICE+KNN) | 5,944,065 | 99,169 | 0 |
| Reimputed MICE | 0 | 263,614 | 0 |
| XGBoost Imputed | 0 | 2,120 | 0 |
| Reimputed XGBoost | 0 | 271 | 0 |
| Median Imputed | 0 | 0 | 0 |

## Supplementary Table (ST4)

**Table ST4.** Reduction in skewness of daily consumption after logarithmic transformation

| **Sample Date** | **Skewness Before** | **Skewness After** |
| --- | --- | --- |
| 01-01-2014 | 62.55 | 0.54 |
| 05-01-2014 | 79.98 | 0.58 |
| 10-02-2014 | 67.32 | 0.47 |
| 15-03-2015 | 88.76 | 0.52 |
| 20-06-2016 | 102.45 | 0.49 |
| 27-10-2016 | 119.03 | 0.24 |
| **Mean Skewness** | **85.67** | **0.48** |

## Supplementary Table (ST5)

**Table ST5.** Ten-fold CV performance of the proposed stacking ensemble model

| **Fold** | **Accuracy (%)** | **Precision (%)** | **Recall (%)** | **F1-Score (%)** | **ROC-AUC (%)** | **Kappa (%)** | **MCC (%)** |
| --- | --- | --- | --- | --- | --- | --- | --- |
| 1 | 94.71 | 93.20 | 96.45 | 94.80 | 98.77 | 89.42 | 89.48 |
| 2 | 94.38 | 92.87 | 96.16 | 94.48 | 98.61 | 88.78 | 88.83 |
| 3 | 94.07 | 92.37 | 96.06 | 94.18 | 98.56 | 88.13 | 88.20 |
| 4 | 94.48 | 93.28 | 95.87 | 94.56 | 98.77 | 88.97 | 89.00 |
| 5 | 94.58 | 92.65 | 96.84 | 94.70 | 98.85 | 89.16 | 89.25 |
| 6 | 94.26 | 92.58 | 96.23 | 94.37 | 98.78 | 88.52 | 88.59 |
| 7 | 94.13 | 92.59 | 95.94 | 94.24 | 98.58 | 88.26 | 88.32 |
| 8 | 94.42 | 93.11 | 95.94 | 94.50 | 98.62 | 88.84 | 88.88 |
| 9 | 94.31 | 93.23 | 95.55 | 94.38 | 98.64 | 88.61 | 88.64 |
| 10 | 94.42 | 92.63 | 96.52 | 94.53 | 98.74 | 88.84 | 88.92 |
| **Mean** | **94.38** | **92.85** | **96.16** | **94.47** | **98.69** | **88.75** | **88.81** |
| **STD** | **0.19** | **0.31** | **0.35** | **0.18** | **0.10** | **0.37** | **0.37** |

## Supplementary Table (ST6)

**Table ST6.**  Performance comparison of STL-Net with stacked ensemble baselines, conventional machine learning models, and deep learning architectures on the SGCC dataset (10-fold stratified CV).

| **Classifier** | **Accuracy (%)** | **Precision (%)** | **Recall (%)** | **F1-Score (%)** | **ROC-AUC (%)** | **MCC (%)** | **Kappa (%)** | **Inference Time (ms/record)** |
| --- | --- | --- | --- | --- | --- | --- | --- | --- |
| **Proposed (STL-Net)** | **94.4** | **92.9** | **96.2** | **94.5** | **98.7** | **88.8** | **88.8** | **6.0** |
| S1– Classical Stacking  (LR meta) | 92.8 | 93.1 | 91.9 | 92.5 | 97.6 | 84.9 | 84.7 | 5.8 |
| S2– Stacking (XGBoost meta) | 94.1 | 94.6 | 93.4 | 94.0 | 98.4 | 87.9 | 87.8 | 7.2 |
| S3–Literature-Inspired Stacking | 91.6 | 91.0 | 92.3 | 91.6 | 97.1 | 83.2 | 83.0 | 6.9 |
| Extra Trees | 94.0 | 92.0 | 94.0 | 93.0 | 98.3 | 87.9 | 87.9 | 2.5 |
| RF | 91.7 | 91.3 | 92.2 | 91.8 | 97.1 | 83.5 | 83.5 | 2.0 |
| KNN | 84.3 | 76.90 | 84.3 | 80.4 | 94.0 | 71.3 | 68.5 | 5.5 |
| CNN | 81.0 | 82.0 | 81.0 | 81.5 | 89.6 | 62.9 | 62.9 | 12.0 |
| DT | 79.9 | 77.8 | 83.5 | 80.6 | 79.9 | 59.9 | 59.7 | 0.8 |
| CNN-LSTM | 73.0 | 73.1 | 73.0 | 73.0 | 81.0 | 46.1 | 46.0 | 25.0 |
| LSTM | 72.5 | 70.0 | 75.0 | 73.0 | 79.3 | 43.5 | 43.5 | 18.0 |
| GBM | 71.5 | 71.7 | 71.5 | 71.6 | 79.0 | 43.2 | 43.1 | 1.8 |
| AdaBoost | 65.0 | 66.0 | 65.0 | 65.5 | 70.6 | 31.1 | 30.8 | 1.5 |
| LR | 62.2 | 62.5 | 62.2 | 62.3 | 67.7 | 24.7 | 24.5 | 0.9 |
| Linear SVM | 62.1 | 62.3 | 62.1 | 62.2 | 67.7 | 24.4 | 24.2 | 1.2 |
| LDA | 62.0 | 60.5 | 69.4 | 64.6 | 67.7 | 24.3 | 24.0 | 0.9 |
| QDA | 58.9 | 56.1 | 82.4 | 66.7 | 65.3 | 20.2 | 17.8 | 1.0 |
| Naive Bayes | 57.7 | 57.7 | 57.7 | 57.6 | 63.5 | 15.3 | 15.3 | 0.9 |

## Supplementary Table (ST7)

**Table ST7**. Statistical comparison of STL‑Net vs. baseline classifiers and stacked ensemble variants

| **Classifier** | **Δ Accuracy (pp)** | **Δ F1-Score (pp)** | **Δ ROC-AUC (pp)** | **95% CI (Accuracy, proportion)** | **Paired *t*-test *p*** |
| --- | --- | --- | --- | --- | --- |
| Extra Trees | 0.38 | 1.50 | 0.39 | [0.0036, 0.0048] | < 1×10⁻⁵ |
| Random Forest | 2.68 | 2.70 | 1.59 | [0.0246, 0.0288] | < 1×10⁻⁵ |
| GBM | 22.88 | 22.90 | 19.69 | [0.2253, 0.2318] | < 1×10⁻⁵ |
| Decision Tree | 14.48 | 13.90 | 18.79 | [0.1418, 0.1481] | < 1×10⁻⁵ |
| KNN | 10.08 | 14.10 | 4.69 | [0.0979, 0.1040] | < 1×10⁻⁵ |
| S1 – Stacking | **–1.62** | **–0.48** | **–0.18** | [–0.0179, –0.0145] | < 1×10⁻⁵ |
| S2 – Stacking | 0.28 | 0.47 | 0.29 | [0.0024, 0.0032] | < 1×10⁻⁵ |
| S3 – Stacking | **–1.73** | **–1.13** | **–0.53** | [–0.0186, –0.0159] | < 1×10⁻⁵ |
| CNN | 13.38 | 13.00 | 9.09 | [0.1308, 0.1372] | < 1×10⁻⁵ |
| CNN-LSTM | 21.38 | 21.50 | 17.69 | [0.2107, 0.2170] | < 1×10⁻⁵ |
| LSTM | 21.88 | 21.50 | 19.39 | [0.2153, 0.2218] | < 1×10⁻⁵ |
| AdaBoost | 29.38 | 29.00 | 28.09 | [0.2908, 0.2971] | < 1×10⁻⁵ |
| Logistic Regression | 32.18 | 32.20 | 30.99 | [0.3182, 0.3247] | < 1×10⁻⁵ |
| Linear SVM | 32.28 | 32.30 | 30.99 | [0.3190, 0.3255] | < 1×10⁻⁵ |
| LDA | 32.38 | 29.90 | 30.99 | [0.3203, 0.3266] | < 1×10⁻⁵ |
| QDA | 35.48 | 27.80 | 33.39 | [0.3512, 0.3575] | < 1×10⁻⁵ |
| Naive Bayes | 36.68 | 36.90 | 35.19 | [0.3634, 0.3699] | < 1×10⁻⁵ |

- Δ values are absolute performance gains of STL-Net over the baseline in percentage points (pp). 95% confidence intervals (CI) are reported for accuracy differences (proportions). All tests are **two-sided**. Family-wise error was controlled using Bonferroni correction (α* ≈ 0.00119).
- Wilcoxon *p*-values = 0.00195 for all models.
- Negative Δ values (S1, S3) indicate cases where STL-Net *slightly underperforms* those stacked baselines on raw accuracy but outperforms them on agreement metrics (Kappa, MCC) and deployment efficiency.
- S2 (XGB-meta stacking) is the strongest competing ensemble, but STL-Net still shows statistically significant gains, validating the benefit of NSGA-II-guided Pareto optimization over accuracy-only stacking.

## Supplementary Table (ST8)

**Table ST8.** Convergence speed, runtime, tuning effort, and implementation complexity (10-fold CV).

| **Classifier** | **Converge time (s)** | **Iterations to Plateau** | **Tuning Effort** | **Implementation Complexity** |
| --- | --- | --- | --- | --- |
| XGBoost | 46 | ~270 | Moderate | Low (standard boosted trees) |
| LightGBM | 35 | ~280 | Moderate | Low (histogram-based trees) |
| CatBoost | 92 | ~1000* | High | Medium (ordered boosting, symmetric trees) |
| NGBoost | 134 | ~240 | High | High (probabilistic + base learner) |
| Proposed  (STL-Net) | 198 | N/A (meta-level CV, 10-fold) | Very High | High (multi-model + meta-learner) |

Notes:

• Iterations to Plateau = first iteration *t* where the 50-round moving-average loss improvement falls below 1×10^-4^

• Hardware: Intel Xeon (Google Colab Pro), CPU only (no GPU).

• Tuning effort bins: Low < 50, Moderate 50–200, High > 200 total evaluations.

• * CatBoost ~1000 iterations from full training logs (not visible in the 0–300 plot).

## Supplementary Table (ST9)

**Table ST9.**  Error-budget decomposition (normalized units). Total Error = Bias + Variance + Irreducible; values normalized to 1 for comparability.

| **Model** | **Bias** | **Variance** | **Irreducible** | **Total Error** |
| --- | --- | --- | --- | --- |
| XGBoost | 0.38 | 0.29 | 0.24 | 0.91 |
| LightGBM | 0.37 | 0.27 | 0.25 | 0.89 |
| CatBoost | 0.31 | 0.34 | 0.23 | 0.88 |
| NGBoost | 0.33 | 0.36 | 0.23 | 0.92 |
| **Proposed (STL‑Net)** | **0.28** | **0.31** | **0.21** | **0.80** |

## Supplementary Table (ST10)

**Table ST10.** Performance under extreme class imbalance versus baseline (fraud class).

| **Classifier** | **ROC-AUC (Imbalanced)** | **Fraud Recall (Imbalanced)** | **Fraud F1 (Imbalanced)** | **Fraud Recall (Balanced)** | **Fraud F1 (Balanced)** | **ΔRecall** | **ΔF1** |
| --- | --- | --- | --- | --- | --- | --- | --- |
| XGBoost | 0.764 | 0.157 | 0.243 | 0.894 | 0.830 | -0.737 | -0.586 |
| LightGBM | 0.778 | 0.117 | 0.191 | 0.865 | 0.801 | -0.748 | -0.610 |
| CatBoost | 0.799 | 0.134 | 0.219 | 0.888 | 0.854 | -0.754 | -0.635 |
| NGBoost | 0.759 | 0.024 | 0.046 | 0.832 | 0.795 | -0.808 | -0.749 |

Notes: Metrics are reported as proportions in [0,1]. Δ Recall and Δ F1 denote absolute change (imbalanced − balanced); negative values indicate degradation.

## Supplementary Table (ST11)

**Table ST11.** Baseline vs temporal-shift performance (train: early period; test: Oct 2016).

| **Metric** | **Baseline** | **Temporal Shift** | **Δ (Temporal)** |
| --- | --- | --- | --- |
| **ROC-AUC** | 0.9869 | 0.9969 | **+0.0100** |
| **Fraud Recall** | 0.8938 | 0.4335 | **-0.4603** |
| **Fraud F1** | 0.8296 | 0.6048 | **-0.2248** |
| Non-Fraud F1 | 0.9693 | 0.9944 | **+0.0251** |
| **Macro F1** | 0.8994 | 0.7996 | **-0.0998** |

Notes: Metrics are proportions. **Δ (Temporal)** = (temporal-shift − baseline); signed change.

**Supplementary Table (ST12)**

**Table ST12**. Comparison of baseline vs noise-injected test performance (Gaussian noise: μ = 0, σ = 0.05).

| **Metric** | **Baseline** | **Noise Test** | **Δ (Noise) (abs)** |
| --- | --- | --- | --- |
| **ROC-AUC** | 0.9869 | 0.9984 | **+0.0115** |
| **Fraud Recall** | 0.8938 | 0.4206 | **-0.4732** |
| **Fraud F1** | 0.8296 | 0.5921 | **-0.2375** |
| Non-Fraud F1 | 0.9693 | 0.9942 | **+0.0249** |
| **Macro F1** | 0.8994 | 0.7932 | **-0.1062** |

Notes: Metrics are proportions. **Δ (Noise)** = (noise − baseline); signed change.

## Supplementary Figures

## Supplementary Figure (SF1)

**
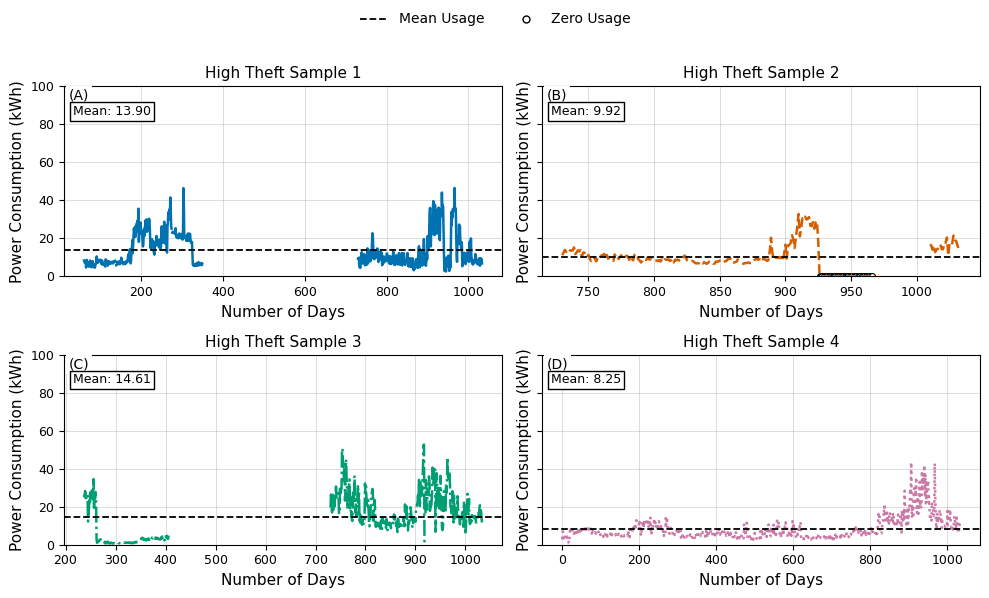
**

**Figure SF1.** Daily consumption patterns of fraudulent consumers with statistical overlays.

## Supplementary Figure (SF2)


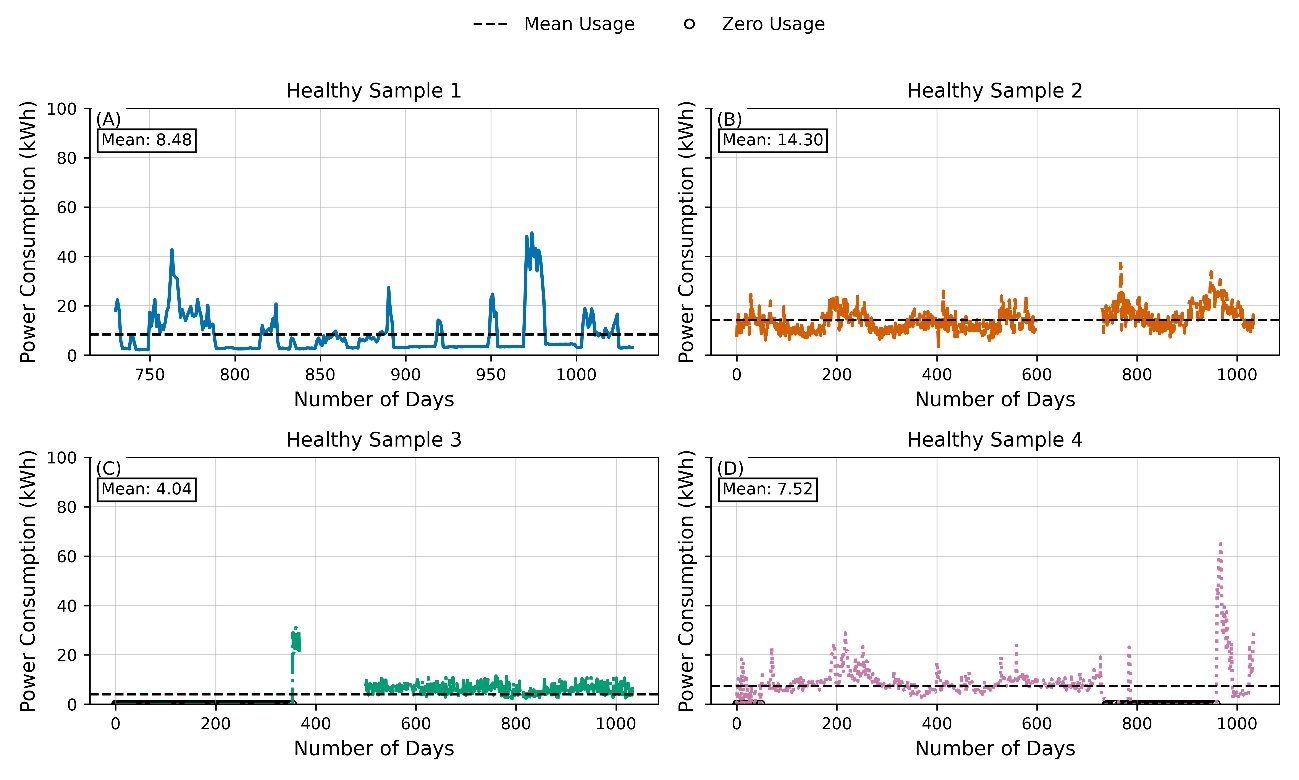


**Figure SF2.** Daily consumption patterns of genuine consumers with statistical overlays.

## Supplementary Figure (SF3)


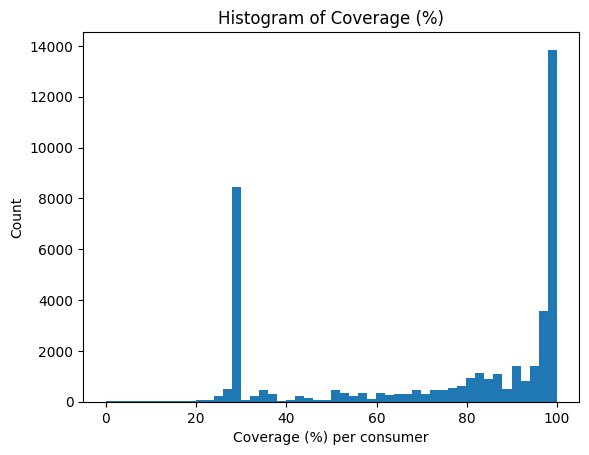


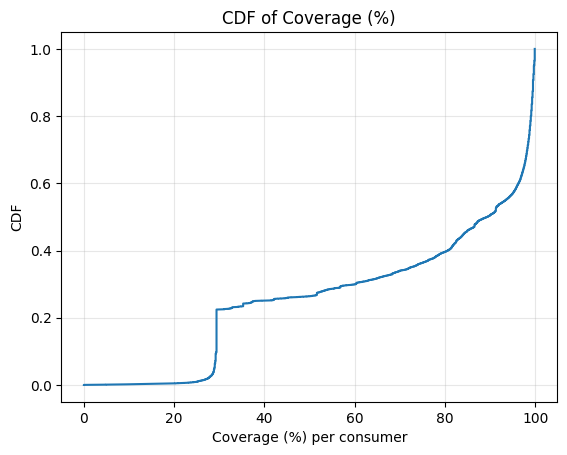


**Figure SF3.** Histogram and CDF of data coverage (%) per consumer

## Supplementary Figure (SF4)


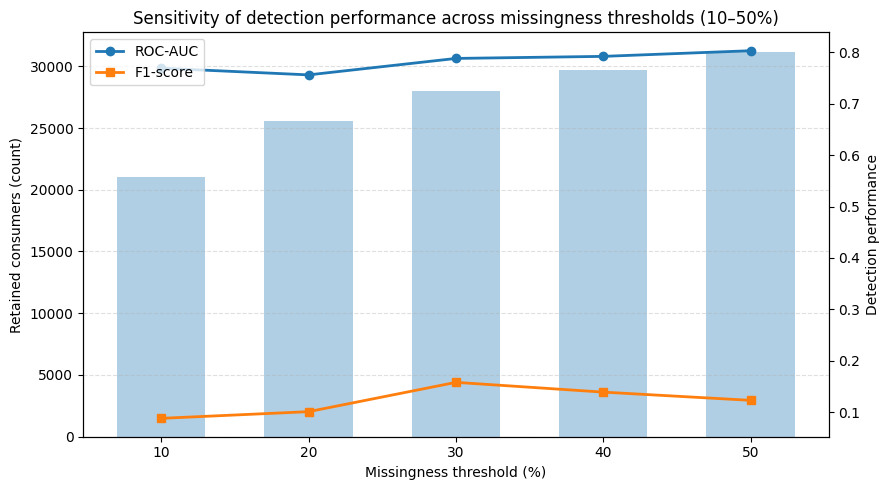


**Figure SF4.** Sensitivity of detection performance across missingness thresholds (10–50%).

## Supplementary Figure (SF5)


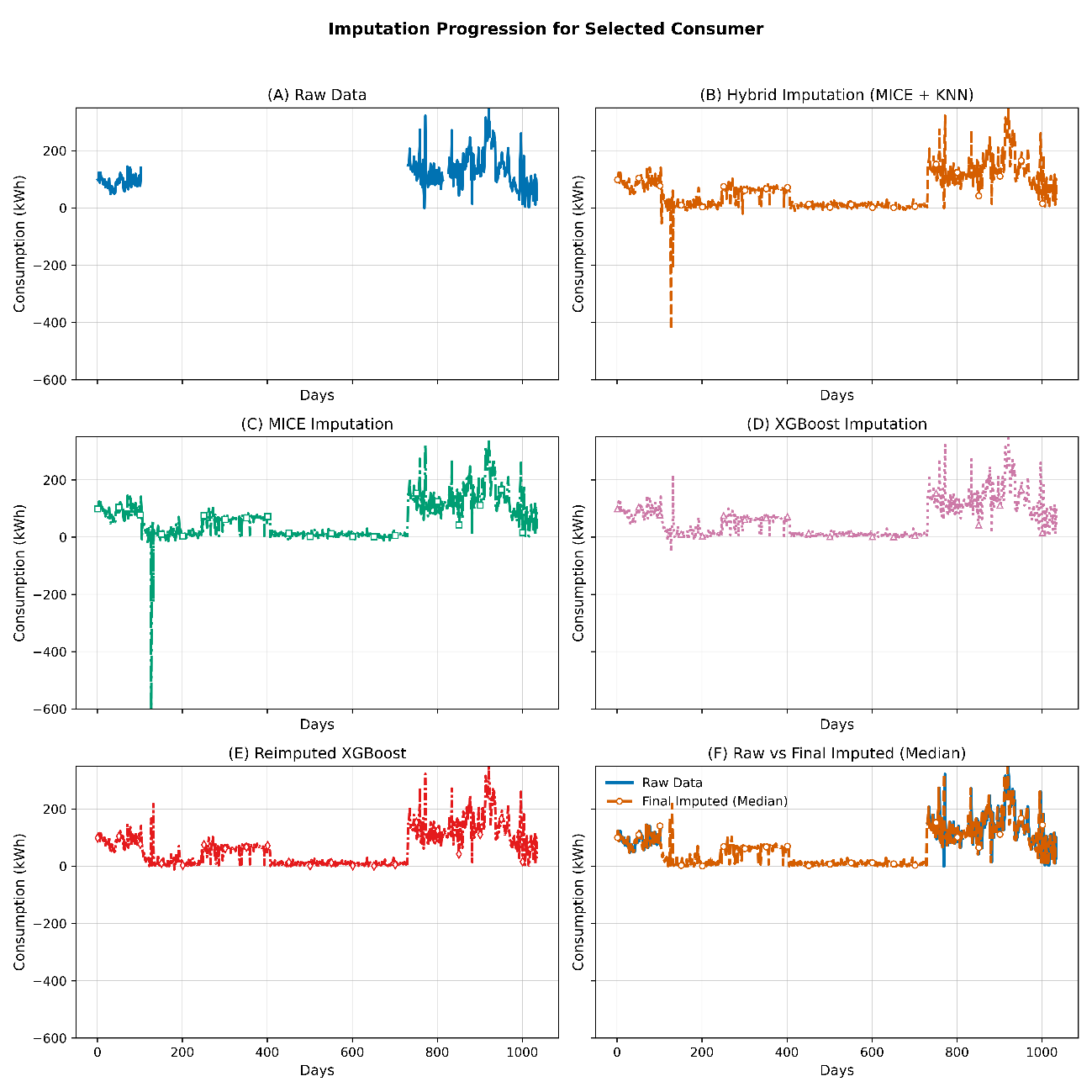


**Figure SF5.** Visual comparison of consumption patterns for a sample consumer before and after each imputation stage.

## Supplementary Figure (SF6)


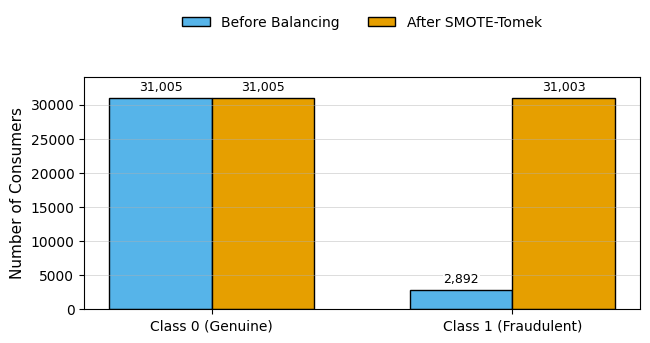


**Figure SF6.** Class distribution before and after SMOTE-Tomek, showing a reduction of class imbalance that can bias classifiers toward the majority class.

## Supplementary Figure (SF7)


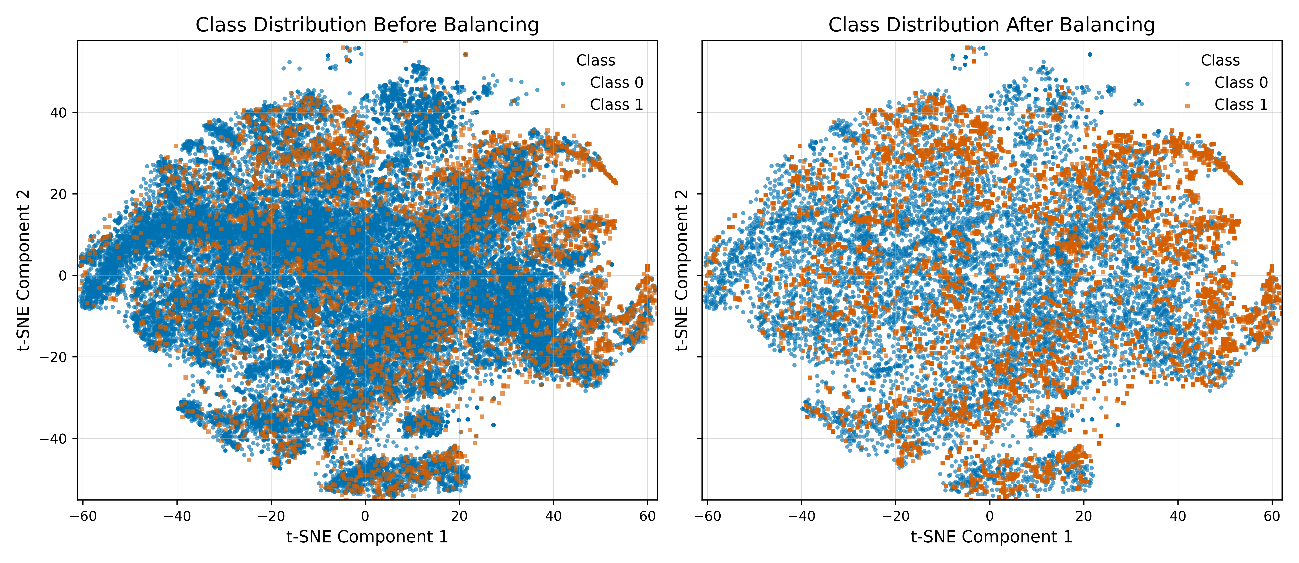


**Figure SF7.**  t-SNE visualization of electricity consumption data before and after SMOTE-Tomek balancing, illustrating enhanced separation between minority and majority classes.

## Supplementary Figure (SF8)


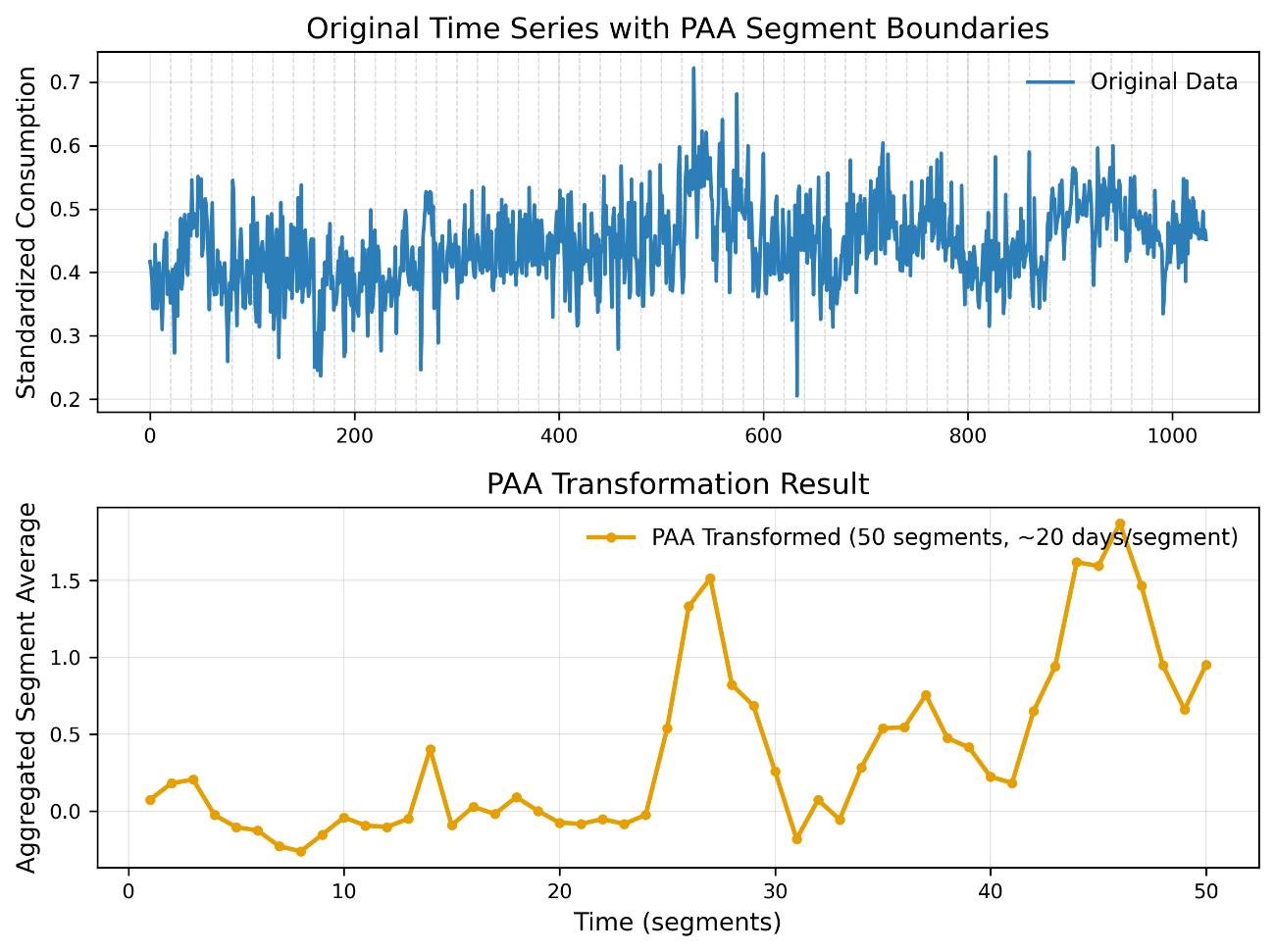


**Figure SF8.** Original standardized electricity consumption time series (top) with 50 segment boundaries (~20 days each) and its Piecewise Aggregate Approximation (PAA) representation (bottom) showing aggregated segment averages.

## Supplementary Figure (SF9)


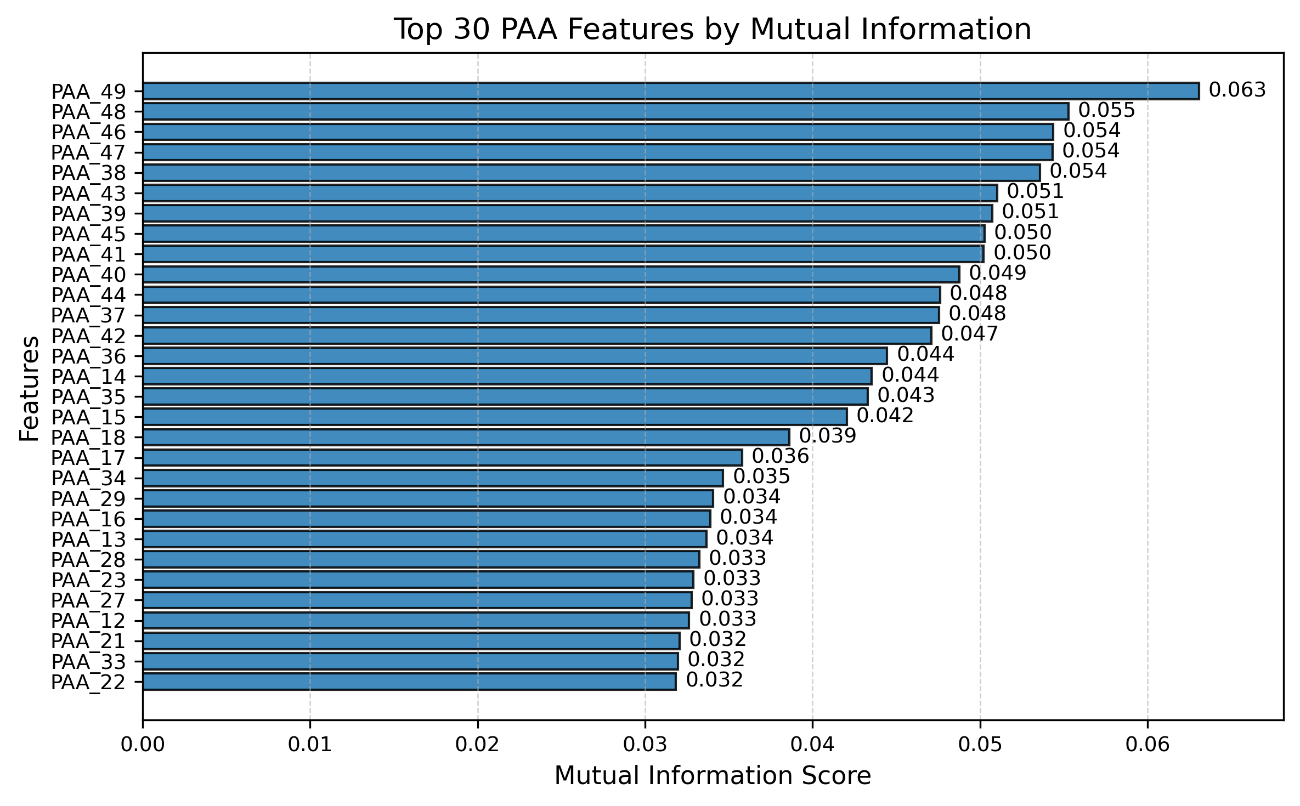


**Figure SF9.** Top 30 PAA features ranked by their MI scores with the target label, computed using mutual_info_classif from scikit-learn.

## Supplementary Figure (SF10)


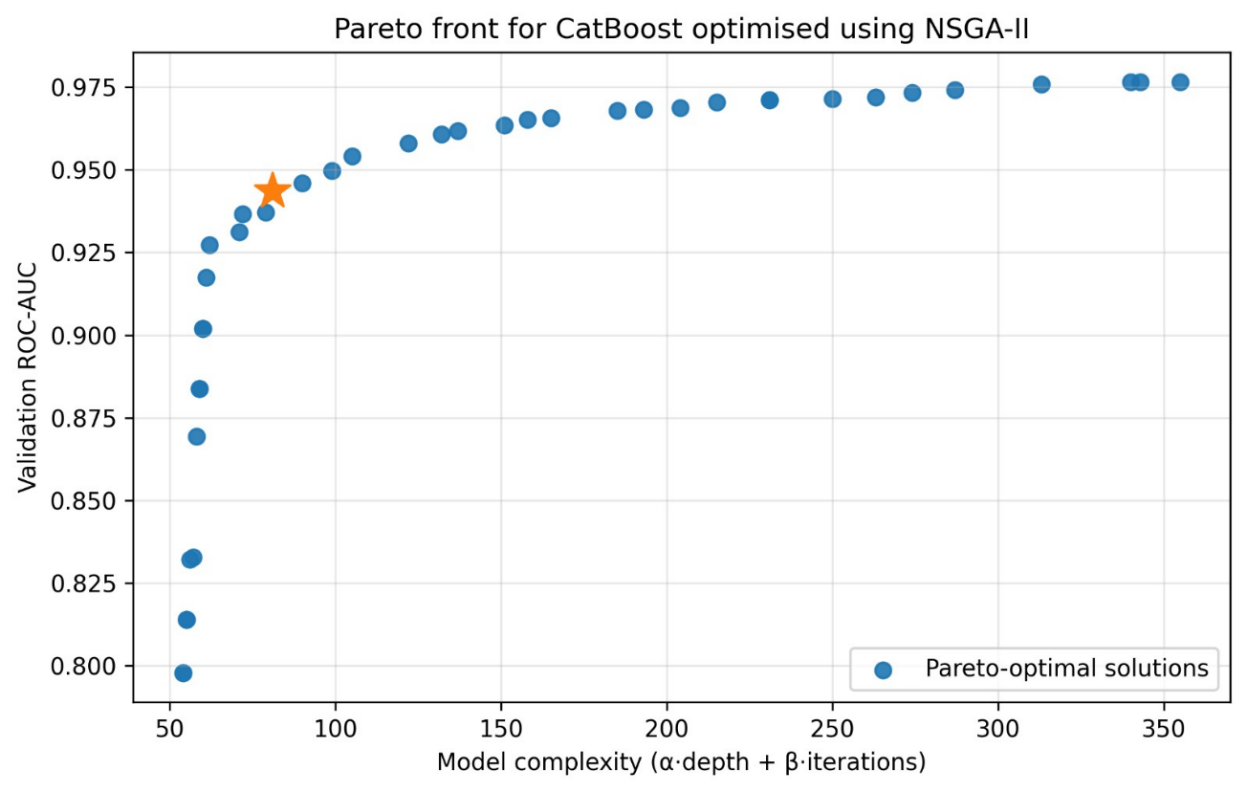


**Figure SF10.** Pareto front obtained using NSGA-II for CatBoost, illustrating the trade-off between validation ROC-AUC and model complexity.

## Supplementary Figure (SF11)


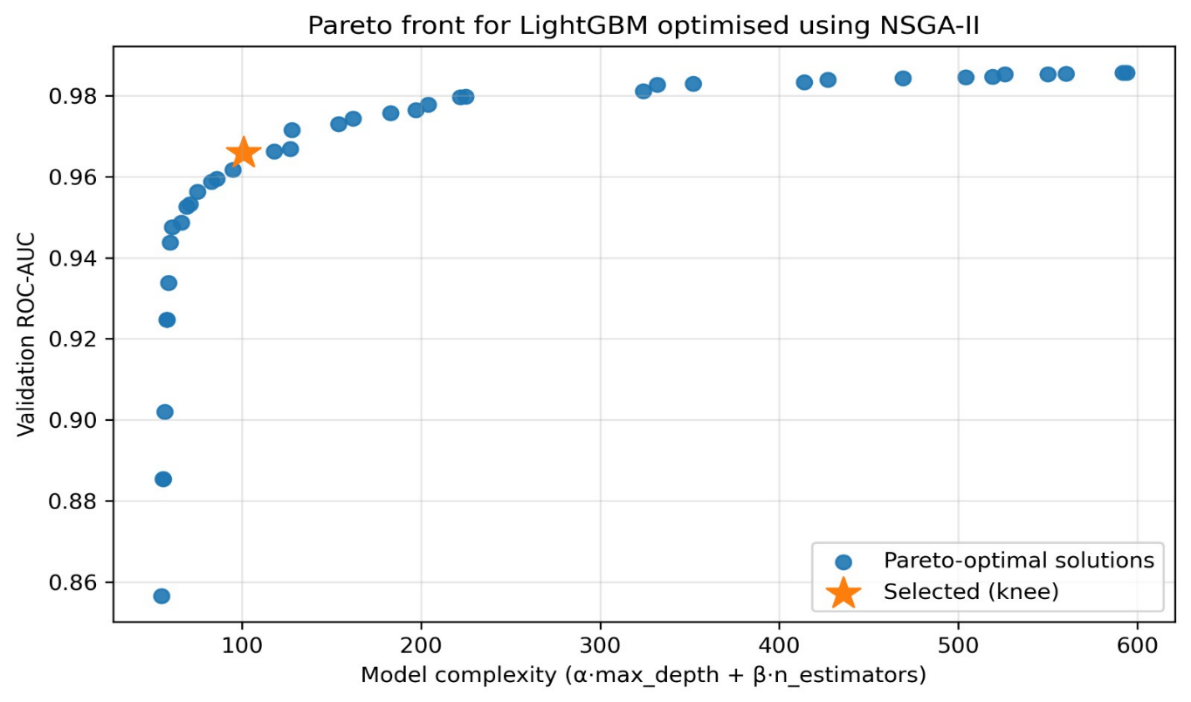


**Figure SF11.** Pareto front obtained using NSGA-II for LightGBM, illustrating the trade-off between validation ROC-AUC and model complexity.

## Supplementary Figure (SF12)


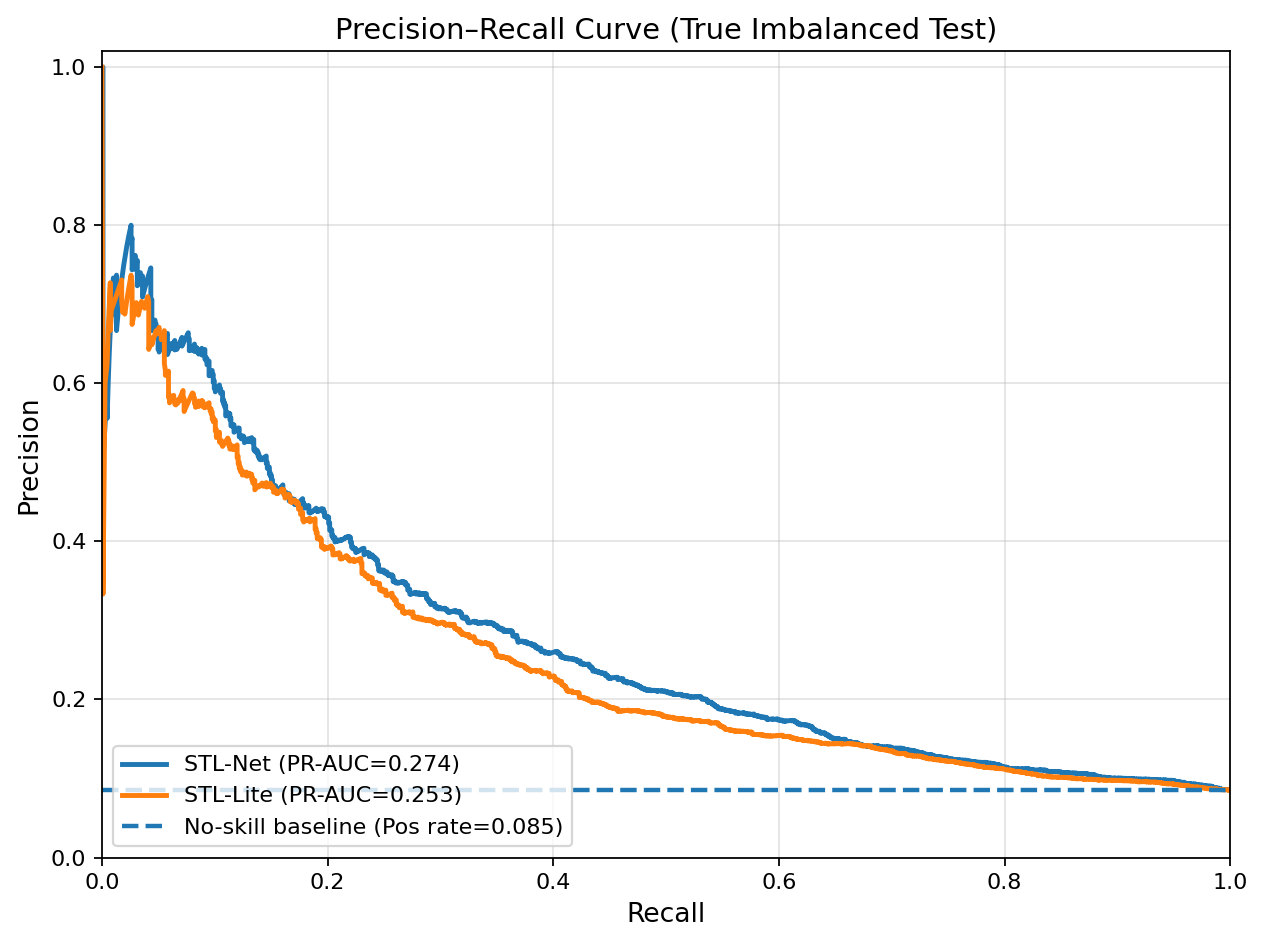


**Figure SF12.** Precision–recall curves for STL-Net and STL-Lite on the true imbalanced test set. The dashed line denotes the no-skill baseline (positive prevalence ≈ 0.085).

## Supplementary Figure (SF13)


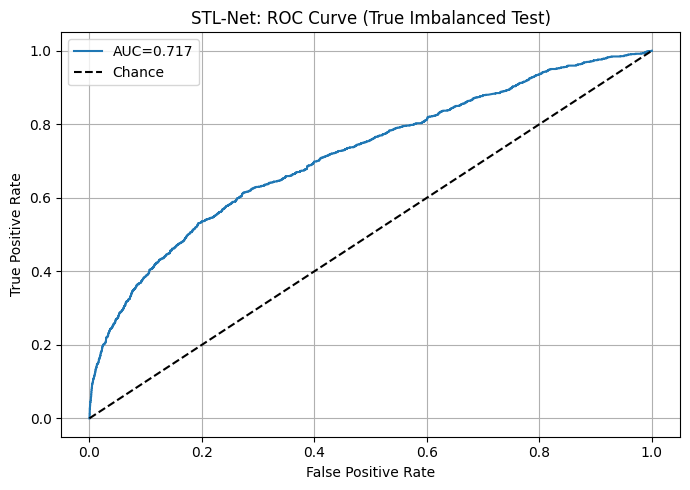


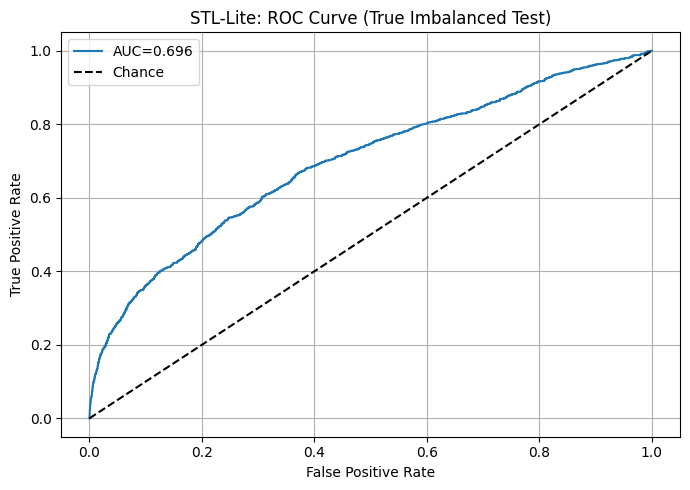


**Figure SF13.** ROC curves for STL-Net and STL-Lite on the true imbalanced test set.
